# IL6 supports long-term expansion of hepatocytes in vitro

**DOI:** 10.1038/s41467-022-35167-8

**Published:** 2022-11-29

**Authors:** Ren Guo, Mengmeng Jiang, Gang Wang, Bing Li, Xiaohui Jia, Yan Ai, Shanshan Chen, Peilan Tang, Aijie Liu, Qianting Yuan, Xin Xie

**Affiliations:** 1 State Key Laboratory of Drug Research, National Center for Drug Screening, Shanghai Institute of Materia Medica, Chinese Academy of Sciences, Shanghai, 201203 China; 2 School of Life Science and Technology, ShanghaiTech University, Shanghai, 201210 China; 3 University of Chinese Academy of Sciences, No.19 A Yuquan Road, 100049 Beijing, China; 4 Department of Pharmaceutics, School of Pharmacy, Fudan University, Shanghai, 201203 China; 5 School of Pharmaceutical Science and Technology, Hangzhou Institute for Advanced Study, University of Chinese Academy of Sciences, Hangzhou, 310024 China; 6 School of Pharmaceutical Science, Nanchang University, Nanchang, 330006 China; 7 Shandong Laboratory of Yantai Drug Discovery, Bohai Rim Advanced Research Institute for Drug Discovery, Yantai, 264117 Shandong China

**Keywords:** Reprogramming, Cell growth, Regeneration

## Abstract

Hepatocytes are very difficult to expand in vitro. A few studies have demonstrated that chemical cocktails with growth factors or Wnt ligands can support long-term expansion of hepatocytes via dedifferentiation. However, the culture conditions are complex, and clonal expansion of hepatic progenitors with full differentiation capacity are rarely reported. Here, we discover IL6, combined with EGF and HGF, promotes long-term expansion (>30 passages in ~150 days with theoretical expansion of ~10^35^ times) of primary mouse hepatocytes in vitro in simple 2D culture, by converting hepatocytes into induced hepatic progenitor cells (iHPCs), which maintain the capacity of differentiation into hepatocytes. IL6 also supports the establishment of single hepatocyte-derived iHPC clones. The summation of the downstream STAT3, ERK and AKT pathways induces a number of transcription factors which support rapid growth. This physiological and simple way may provide ideas for culturing previously difficult-to-culture cell types and support their future applications.

## Abstract (web summary)

Hepatocytes are very difficult to expand in vitro. Here the authors discover that IL6 promotes long-term expansion (>30 passages) of primary mouse hepatocytes in vitro by converting the cells into hepatic progenitor cells, which maintain full capacity of differentiation into hepatocytes.

## Introduction

Liver is the primary metabolic organ in mammals. During homeostasis, the liver cells generally have a low turnover rate^1^. However, liver exhibits robust regeneration following chemical, viral or mechanical injuries, attributing to the immediate proliferation of hepatocytes^1–4^. After 2/3 partial hepatectomy (PHX) in mice, it takes only 1 week for the remaining liver to regenerate to the original size^5^. Comparing to the robust proliferation capacity in vivo, adult hepatocytes have been proven to be extremely difficult to culture and expand without losing their hepatic characteristics in vitro^6,7^. Due to the wide application of primary hepatocytes in drug toxicity and metabolism evaluation, and the potential use of hepatocyte cell therapy in liver diseases, to identify ways to expand hepatocytes in vitro is an urgent task.

It has been reported that hepatocyte replenishment following liver damage occurs via a reversible transition between hepatocytes and duct-like progenitor cells^8^. In 2017, two studies opened up a new dimension in hepatocytes culture following this progenitor idea. One has reported that a cocktail of small molecules A-83-01 (inhibitor of TGF-β signaling), Y27632 (inhibitor of ROCK kinase) and CHIR99021 (activator of canonical Wnt pathway) can convert rat and mouse hepatocytes into hepatic progenitor cells (HPC) with high proliferative capacity^9^. Another study has shown that a combination of five molecules A-83-01, Y27632, CHIR99021, S1P (sphingosine-1-phosphate) and LPA (lysophosphatidic acid) can expand mouse hepatocytes in vitro by promoting a reversible transition between hepatocytes and HPC^10^. Interestingly, a study has reported the combination of TNFα, (a vital inflammatory cytokine in liver regeneration^1^), A-83-01, Y27632 and CHIR99021 could promote mouse hepatocyte proliferation in 3D culture^11^. After that, a protocol that enables large-scale expansion of human hepatocytes, by using Wnt3a protein with A-83-01 and Y27632 in low oxygen condition, has been reported^12^. Recently more studies reported the culture of human fetal or adult hepatocytes using chemicals in combination with growth factors or conditioned medium in 2D or 3D conditions^13–16^. These conditions (Supplementary Table 1) all involve the use of chemicals which do not exist in vivo. And clonal expansion from a single liver/hepatocyte progenitor cell with full differentiation capacity are only seen in rat hepatocytes^9^. Is there a more physiological and simple way that can support clonal expansion of hepatocytes?

In partial hepatectomy model, many signaling molecules, including EGF, HGF, IL6, TNFα, insulin, bile acids, leptin, etc., increase rapidly in blood after the surgery^1,17–20^. IL6 is a cytokine widely studied in inflammation. Strikingly, IL6 also plays a central role in liver regeneration^1,19,20^. After partial hepatectomy, IL6 is rapidly produced by both Kupffer cells and hepatocytes^21,22^. In IL6 knockout mice, liver regeneration delays accompanied by increased liver necrosis after partial hepatectomy^21,23^, which could be corrected by IL6 treatment^23^. IL6 knockout increases hepatocytes apoptosis following CCl_4_ treatment, whereas pretreatment with IL6 reduces liver injury and accelerate regeneration^23,24^. Similar phenomena have also been observed in IL6 receptor deficient mice^25^. Although many evidences support that IL6 promotes liver regeneration in vivo, whether it can be used to support long-term expansion of hepatocytes in vitro remain unclear.

Here, we report that IL6 could promote long-term expansion (>30 passages in ~150 days with theoretical expansion of ~10^35^ times) of primary mouse hepatocytes in vitro, by converting hepatocytes into induced hepatic progenitor cells (iHPCs) which maintain the capacity of differentiation into hepatocytes. IL6 also supports the establishment of single hepatocyte-derived iHPC clones. Hepatocytes differentiated from these iHPCs could easily engraft and repopulate the whole liver of *Fah*^−/−^ mice. Further studies show IL6 mainly promotes the proliferation of diploidy hepatocytes. And a number of transcription factors, including Barx2, FoxM1, Elf3 and Mxd3, plays critical roles in IL6 induced hepatocytes proliferation.

## Results

### IL6 promotes primary hepatocytes expansion in vitro

**Fig. 1:**
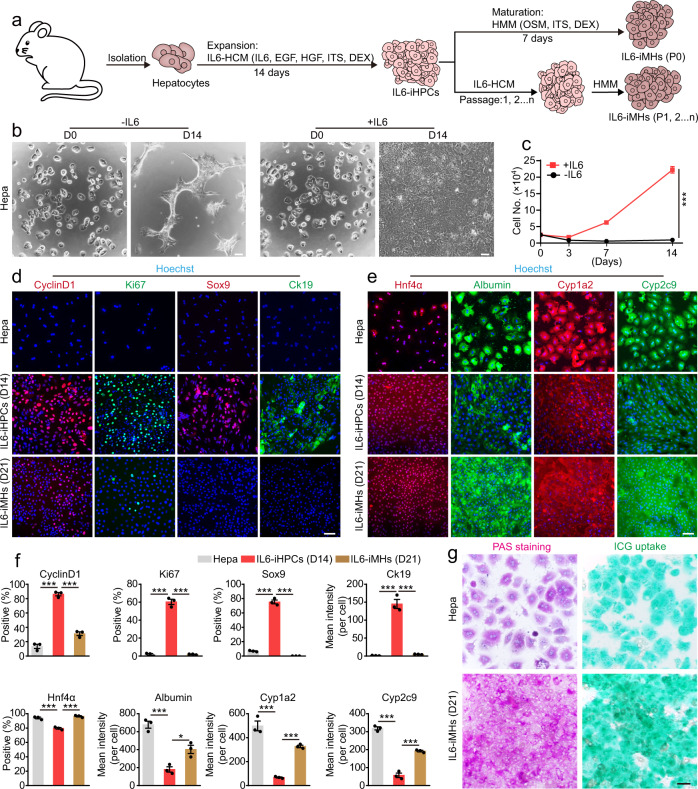
IL6 promotes primary hepatocytes expansion in vitro. **a** Schematic of IL6-induced hepatocyte to iHPC reprogramming and subsequent maturation to iMHs. **b** Representative images of the primary hepatocytes (Hepa, D0) and hepatocytes cultured with or without IL6 for 14 days (D14) (repeated for three times). **c** Growth curves of primary hepatocytes cultured with or without IL6 for 14 days. Data are Means ± SEM (*n* = 3 independent experiments). ***p < 0.001 (two-way ANOVA). Immunofluorescence staining of cell cycle markers CyclinD1 (red) and Ki67 (green), HPC markers Sox9 (red) and Ck19 (green) (**d**) and hepatic markers Hnf4α (red) Albumin (green), Cyp1a2 (red) and Cyp2c9 (green) (**e**) in primary hepatocytes (Hepa), IL6-iHPCs (D14) and IL6-iMHs (D21). Nuclei were stained with Hoechst 33342 (blue). **f** Statistical data of the immunofluorescence staining data in **d** and **e**. Data are Means ± SEM (Cells from three mice were analyzed independently, three random fields/mouse). *p < 0.05, ***p < 0.001 (two-tailed unpaired Student’s *t* test). **g** Representative images of PAS staining and ICG uptake in primary hepatocytes and IL6-iMHs (D21) (repeated for three times). Scale bars represent 100 µm. DEX dexamethasone, OSM oncostatin M. Source data and the exact *p* values are provided in a Source data file.

Previous studies have shown that IL6 and TNFα are the most important inflammatory cytokines involved in liver regeneration in vivo^1,19,20^. To test their effects on hepatocyte proliferation, we cultured hepatocytes in the classical hepatocyte culture medium (HCM, containing epidermal growth factor (EGF) and hepatocyte growth factor (HGF)) supplemented with IL6 or TNFα. Consist with the general notion^9,26^, no proliferation of hepatocytes was observed in HCM at day 14 (Supplementary Fig. 1a, b). TNFα slightly promoted cell growth, while IL6 stimulated significant cell proliferation (Supplementary Fig. 1a, b). More concentrations of IL6 were tested, significant growth of primary hepatocytes could be observed with 0.1 ng/ml IL6, and growth reached a plateau at 3 ng/ml (Supplementary Fig. 1c, d). We passaged the hepatocytes in media containing 0.3–30 ng/ml IL6 at D14 and cultured them again in the same media with the same starting number, and only the higher concentrations (10 and 30 ng/ml) supported further rapid growth (Supplementary Fig. 1e). So, we used 30 ng/ml IL6 for further studies. The growth stimulating effect of IL6 could be blocked by IL6-antibody in a dose-dependent manner (Supplementary Fig. 2a–d). Previous studies have shown that EGF and HGF are important components of the early signaling pathways leading to liver regeneration/hepatocyte proliferation in vivo^1,19^. And EGF and HGF are widely used to culture primary hepatocytes^10,12^. Here we found EGF and HGF were not enough the stimulate the growth of hepatocytes, similar to previous studies^10^, but either one in combination of IL6 powerfully stimulated the growth of hepatocytes, and the combination of three gave the best result (Supplementary Fig. 2e, f). Based on these results, we summarized a strategy for using IL6 to promote hepatocytes proliferation in vitro (Fig. 1a), and the expansion medium was named as IL6-HCM. The proliferating cells at D14 shown typical epithelial morphology with a high nucleus/cytoplasm ratio, which is a typical feature of HPC^9^ (Fig. 1b). Comparing to freshly isolated hepatocytes, the cells cultured in IL6-HCM (D14) grow rapidly (Fig. 1c) and were highly positive for cell cycle markers Cyclin D1 and Ki67, and HPC markers Sox9 and Ck19, but the genes related to the functions of hepatocytes, including Albumin, Hnf4α, Cyp1a2 and Cyp2c9, were significantly reduced (Fig. 1d–f). So, these cells were named IL6-induced HPCs (IL6-iHPCs).

IL6-iHPCs (D14) could be differentiated into mature hepatocytes using a widely used hepatic maturation medium (HMM)^9^ with minor modifications (Fig. 1a). After culturing IL6-iHPCs (D14) in HMM for 7 days, the cells differentiate into typical mature hepatocyte morphology (Supplementary Fig. 3a), we named these cells as IL6-induced mature hepatocytes (IL6-iMHs). Immunofluorescence staining revealed that IL6-iMHs expressed low level of cell cycle markers CyclinD1 and Ki67, almost no HPC markers Sox9 and Ck19, and the genes highly expressed in mature hepatocytes including Hnf4α, Albumin, Cyp1a2 and Cyp2c9 were significantly increased to levels comparable to primary hepatocytes (Fig. 1d–f). Quantitative RT-PCR analysis also confirmed more HPC genes and cell cycle genes were upregulated in IL6-iHPCs (D14), but greatly reduced in IL6-iMHs, while the genes related to mature hepatocyte functions were all downregulated in IL6-iHPCs (D14), but then upregulated in IL6-iMHs (Supplementary Fig. 3b). Periodic acid-Schiff (PAS) and indocyanine green (ICG) uptake assay also showed that IL6-iMHs possessed the mature hepatocytes functions (Fig. 1g). Taken together, we found that IL6 could reprogram primary hepatocytes into iHPCs with high proliferation ability, and the IL6-iHPCs could be differentiated into mature hepatocytes.

### Long-term expansion of IL6-iHPCs with full differentiation capacity

**Fig. 2:**
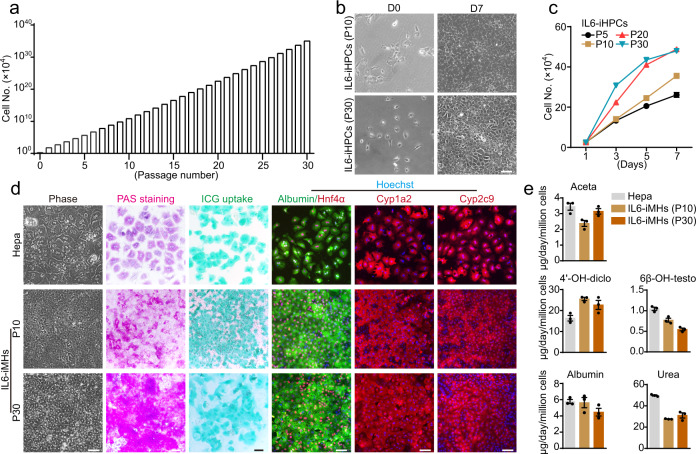
IL6 support long-term expansion of iHPCs without losing the differentiation capacity. **a** Calculated cumulative cell numbers of IL6-iHPCs for 30 passages. **b** Representative images of IL6-iHPCs (P10 and P30) cultured with IL6 at D0 and D7. **c** The growth curve of IL6-iHPCs from different passages. Data are Means ± SEM (*n* = 4 independent experiments). **d** Representative phase contrast images, PAS staining, ICG uptake and immunofluorescence staining of hepatic markers Albumin (green), Hnf4α (red), Cyp1a2 (red) and Cyp2c9 (red) in IL6-iMHs (P10 and P30), primary hepatocytes were used as control. Nuclei were stained with Hoechst 33342 (blue). **e** Albumin and urea secretion, and drug (phenacetin, diclofenac and testosterone) metabolism of IL6-iMHs (P10) and primary hepatocytes. The metabolic products (acetaminophen (Aceta), 4’-OH-diclofenac (4’-OH-diclo) and 6β-OH-testosterone (6β-OH-testo)) were measured by liquid chromatography-tandem mass spectrometry. Data are Means ± SEM (*n* = 3 independent experiments), normalized by cell numbers. Scale bars represent 100 µm. Source data are provided in a Source data file.

To test whether IL6 can support long-term expansion of iHPCs, IL6-iHPCs were passaged every 5–7 days (∼90% confluence). IL6-iHPCs could be passaged for more than 30 times and the cumulative cell number increased from 2.5 × 10^4^ to about 1 × 10^40^ in ~150 days without any apparent morphological changes (Fig. 2a, b and Supplementary Fig. 4a). Interestingly, the growth rate kept increasing till reach a plateau at passage 20 (P20) (Fig. 2c). So, the time between passages gradually reduce from 7 to 5 days. Immunofluorescence staining revealed that IL6-iHPCs at P5, P10, P20 and P30 showed stable and high expression of the cell cycle markers CyclinD1 and Ki67 and hepatic progenitor markers Sox9 and Ck19 (Supplementary Fig. 4b, c). IL6-iHPCs at all passages could be differentiated into cells with typical mature hepatocyte-like morphology, these cells were named as IL6-iMHs-P5, P10, P20 and P30, respectively (Fig. 2d and Supplementary Fig. 5a). PAS staining, ICG uptake, immunofluorescence staining of Albumin, Hnf4α, Cyp1a2 and Cyp2c9 showed that IL6-iMHs-P5, P10, P20 and P30 were very similar to primary hepatocytes in these aspects (Fig. 2d and Supplementary Fig. 5a, b). More genes related to mature hepatocytes functions were also highly expressed by IL6-iMHs (Supplementary Fig. 5c). Similar to primary hepatocytes, IL6-iMHs were able to secrete albumin, produce urea and metabolize drugs (Fig. 2e).

### Generation of iHPC clones from single hepatocyte with IL6

**Fig. 3:**
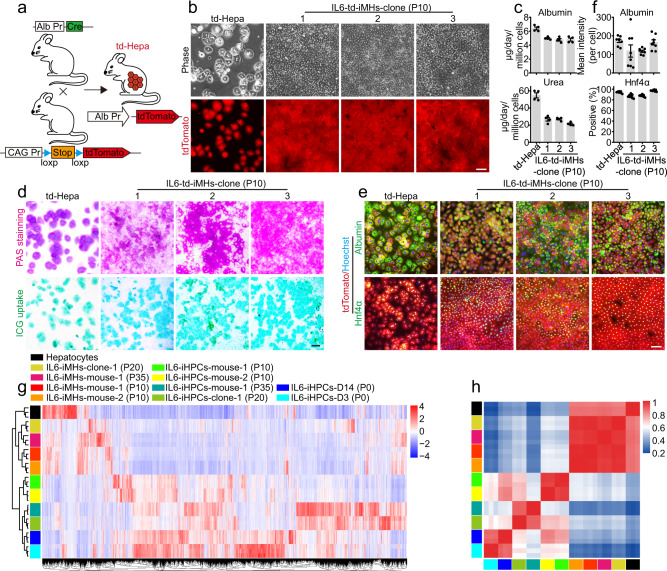
Generation of iHPC clones from single cells with IL6. **a** Schematic diagram of the genetic fate mapping method used to produce mice with hepatocytes expressing tdTomato (td-Hepa). **b** Representative morphologies and tdTomato (red) images of td-Hepa and three IL6-td-iMH clones (P10) derived from single cells. **c** The albumin and urea secretion capacities of IL6-td-iMH clones (P10) and primary td-Hepa. Data are Means ± SEM (*n* = 4 independent experiments). Representative PAS staining, ICG uptake (**d**) and immunofluorescence staining of hepatic markers Albumin (green) and Hnf4α (green) (**e**) in primary td-Hepa and IL6-td-iMH clones (P10). Nuclei were stained with Hoechst 33342 (blue). **f** Statistical data of the immunofluorescence staining data in **e**. Data are Means ± SEM (eight random fields for each group, three independently generated IL6-td-iMH clones were analyzed). **g** Transcriptome analysis among primary hepatocytes, IL6-iHPCs-D3 (P0), IL6-iHPCs-D14 (P0), IL6-iHPCs from different mouse and different passages and the IL6-iMHs differentiated from them (*n* = 3 technical repeats). The color bar at the right indicates gene expression in log2 scale. Red and green represent higher and lower gene expression levels respectively. **h** Pearson correlation map of primary hepatocytes, IL6-iHPCs-D3 (P0), IL6-iHPCs-D14 (P0), IL6-iHPCs from different mouse and different passages and the IL6-iMHs differentiated from them. The color bar at the right indicates a value of correlation between two samples. Red and blue colors represent higher or lower correlation coefficient between samples (*n* = 3 technical repeats). Scale bars represent 100 µm. Source data are provided in a Source data file.

To confirm that IL6-iHPCs were indeed arise from hepatocytes, but not existing progenitor cells, a lineage-tracing experiment was carried out. R26R^tdTomato^ mice, in which tdTomato expression is prevented by a loxp-flanked STOP cassette, were crossed with the *Albumin*-Cre mice^27^ and the offspring (Alb-td-mice) would have the tdTomato expressing in all hepatocytes (td-Hepatocytes) specifically (Fig. 3a). Similar to our previous findings, td-Hepatocytes could also be reprogrammed into tdTomato^+^ IL6-iHPCs (IL6-td-iHPCs) (Supplementary Fig. 6a). Furthermore, we successfully established three single td-Hepatocyte-derived IL6-iHPCs lines (IL6-td-iHPCs-clone-1, 2 and 3, from three mice), indicating the powerful growth promotion effect of IL6. As expected, IL6-td-iHPCs-clones all possessed high proliferative capacity in IL6-HCM (Supplementary Fig. 6b–d) and were highly positive for cell cycle markers CyclinD1 and Ki67 and hepatic progenitor markers Sox9 and Ck19 (Supplementary Fig. 6e, f, i). These clones could also be differentiated into mature hepatocytes (IL6-td-iMHs-clone-1, 2 and 3) (Fig. 3b). The albumin and urea secretion (Fig. 3c), PAS staining, ICG uptake (Fig. 3d) and immunofluorescence staining of hepatic markers Albumin, Hnf4α, Cyp1a2 and Cyp2c9 showed that IL6-td-iMHs-clone-1, 2 and 3 were comparable to td-Hepatocytes (Fig. 3e, f and Supplementary Fig. 6g–i). Moreover, these IL6-td-iHPCs-clones could be passaged for at least 30 times (Supplementary Fig. 6j), and could still be differentiated into mature hepatocytes with high expression of Albumin and Hnf4α (Supplementary Fig. 6k).

For further characterization, transcriptomic comparison was carried out among primary hepatocytes, IL6-iHPCs-D3 (P0), IL6-iHPCs-D14 (P0), IL6-iHPCs from different mouse and different passages and the IL6-iMHs differentiated from them (Fig. 3g, h). The profiles of primary hepatocytes and IL6-iMHs were highly similar and they were grouped together. The IL6 treated cells, even only for 3 days, were totally different from primary hepatocytes, indicating a clear reprogramming by IL6 (Fig. 3g, h). The cell cycle genes were dramatically upregulated in IL6-iHPCs but were downregulated in IL6-iMHs (Supplementary Fig. 7a). Genes related to hepatic function, including cytochromes, bile acid biosynthesis and metabolism, glucose metabolism, coagulation factors, lipid and fatty acid metabolism and xenobiotic metabolism, were downregulated in IL6-iHPCs and then upregulated in IL6-iMHs to similar levels as in primary hepatocytes (Supplementary Fig. 7b).

These results confirm that IL6-iHPCs do originate from hepatocytes and IL6 could be used to establish single hepatocyte-derived iHPC clones which retain full hepatic differentiation capacity after serial passages.

### IL6-iMHs repopulate livers of *Fah*^−/−^ mice

**Fig. 4:**
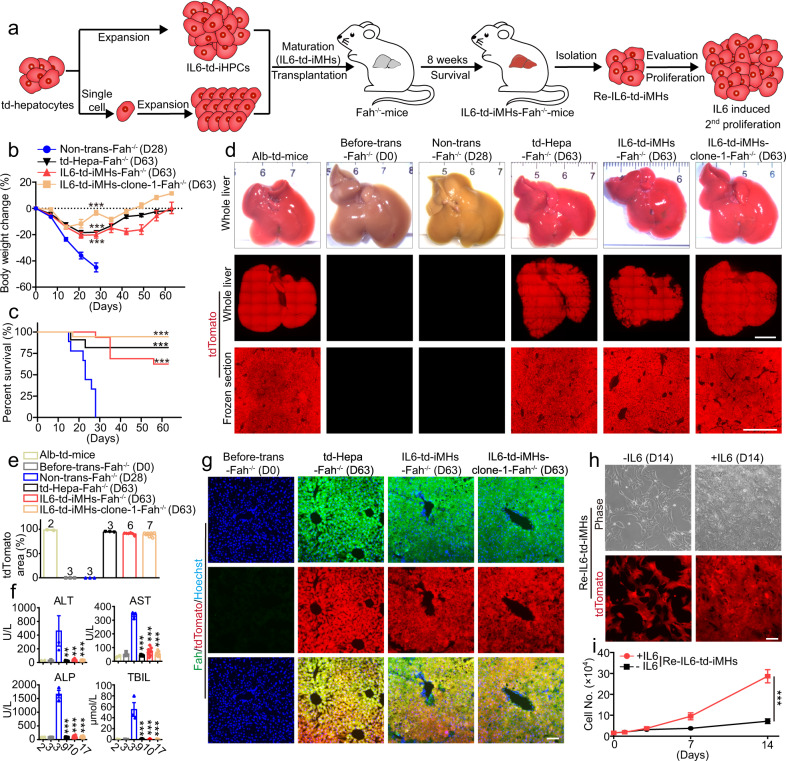
IL6 derived iMHs repopulate livers of Fah^−/−^ mice. **a** Schematic diagram of the experimental procedure: td-iMHs matured from IL6-expanded td-iHPCs (mixed population or single-cell clones) were transplanted into the liver of *Fah*^−/−^ mice, and the repopulated td-iMHs were isolated again 8 weeks later and tested for 2nd round of expansion with IL6. **b** Body weight change of *Fah*^−/−^ mice (no NTBC) receiving vehicle (Non-trans, *n* = 9 mice), primary td-hepatocytes (td-Hepa, *n* = 11 mice), IL6-td-iMHs (P10, *n* = 16 mice) and IL6-td-iMHs-clone-1 (P10, *n* = 18 mice). Data are Means ± SEM. Statistical differences between two groups were analyzed from D0–D28. ***p < 0.001 (two-way ANOVA). **c** Survival curves of the animals in **b**. ***p < 0.001 (Log-rank test). **d** Representative images of the whole livers (top), and fluorescent images of the whole livers (middle, scale bars represent 5 mm) and frozen sections of liver (down, scale bars represent 1 mm) from *Fah*^−/−^ mice before NTBC withdrawal (D0, *n* = 2 mice), 28 days after vehicle transplantation (no NTBC, *n* = 3 mice), or 63 days after td-Hepa (*n* = 3 mice), IL6-td-iMHs (*n* = 6 mice), or IL6-td-iMHs-clone-1 (*n* = 7 mice) transplantation (no NTBC). The liver of Alb-td-mice was used as control. **e** Quantitative analysis of tdTomato positive areas in liver (*n* = 2–7 mice, numbers of the animals were listed on top of the bar). **f** Serum levels of ALT, AST, ALP and TBIL in the *Fah*^−/−^ mice after transplantation. Data are Means ± SEM (*n* = 2–17 mice, numbers of the animals were listed under the bar). **p < 0.01, ***p < 0.001 (two-tailed unpaired Student’s *t* test). **g** Immunofluorescence staining of hepatic marker Fah (green) in frozen liver sections of *Fah*^−/−^ mice before or 63 days after transplantation. Nuclei were stained with Hoechst 33342 (blue). Representative morphology and tdTomato images (**h**) and growth curves (**i**) of hepatocytes isolated from the *Fah*^−/−^ mice repopulated with IL6-td-iMHs for 63 days and then cultured in vitro with or without IL6 for 14 days. Data are Means ± SEM (*n* = 4 independent experiments). ***p < 0.001 (two-way ANOVA). Scale bars represent 100 µm. ALT alanine aminotransferase, AST aspartate aminotransferase, ALP alkaline phosphatase, TBIL total bilirubin. Source data and exact *p* values are provided in a Source data file.

*Fah*^−/−^ mice lack the tyrosine metabolic enzyme fumarylacetoacetate hydrolase (Fah) and require 2-(2-nitro-4-trifluoro-methylbenzyol)−1,3-cyclohexanedione (NTBC) supply for survival^28,29^. After NTBC withdrawal, *Fah*^−/−^ mice undergo liver failure and die in about 30 days, but they can be rescued by transplantation of functional hepatocytes^30–33^. This is an ideal model to evaluate hepatocyte functions in vivo. Thereby, after NTBC withdrawal, td-Hepatocytes, IL6-td-iMHs or IL6-td-iMHs-clone-1 (the iMHs were at P10, cultured in IL6-HCM for ~60 days and then in HMM for another 7 days) were transplanted into *Fah*^−/−^ mice via intrasplenic injection through a left-flank incision under tribromoethanol anesthesia (Fig. 4a). After NTBC withdrawal, all nine *Fah*^−/−^ mice without transplantation showed continuous body weight loss (Fig. 4b) and died within 30 days (Fig. 4c), and their livers showed severe inflammation and necrosis (Fig. 4d and Supplementary Fig. 8a). In contrast, the body weight of *Fah*^−/−^ mice receiving td-Hepatocytes, IL6-td-iMHs or IL6-td-iMHs-clone-1 began to recover after 2–3 weeks of transplantation (Fig. 4b). Remarkable, 9 out of 11 *Fah*^−/−^ mice receiving td-Hepatocytes, 10 out of 16 *Fah*^−/−^ mice receiving IL6-td-iMHs, 17 out of 18 *Fah*^−/−^ mice receiving IL6-iMHs-clone 1 survived for 2 months after NTBC withdrawal (Fig. 4c). Two months after transplantation, the livers receiving td-Hepatocytes, IL6-td-iMHs and IL6-td-iMHs-clone-1 were as normal as the Alb-td-mice and *Fah*^−/−^ mice before NTBC withdrawal/transplantation (Fig. 4d and Supplementary Fig. 8a). IL6-td-iMHs and IL6-td-iMHs-clone-1 could repopulate about 95% of the liver, similar to td-Hepatocytes (Fig. 4d, e and Supplementary Fig. 8b). The highly increased serum levels of AST, ALT, ALP and total bilirubin after NTBC withdrawal were almost returned to normal after transplantation (Fig. 4f). The repopulated td-Hepatocytes, IL6-td-iMHs and IL6-td-iMHs-clone-1 in *Fah*^−/−^ mice were almost 100% positive for Fah, Hnf4α and Albumin staining (Fig. 4g and Supplementary Fig. 8c, d). Interestingly, although IL6-iMHs were matured from IL6-iHPCs which were express the HPC and biliary epithelial cell marker Ck19, the repopulated IL6-td-iMHs were negative for Ck19, indicating no conversion of IL6-iMHs to biliary epithelial cells in vivo (Supplementary Fig. 8e). As previously reported, although hepatocytes appear histologically homogeneous, the liver lobule is actually organized into concentric zones, or rings, in which hepatocytes express different metabolic enzymes across the portal vein to central vein axis through which blood flows^34^. Typically, Glutamine synthetase (GS) is positive in pericentral hepatocytes and Arginase1 (Arg1) is positive in periportal hepatocytes^34,35^. Interestingly, the repopulated td-Hepatocytes, IL6-td-iMHs and IL6-td-iMHs-clone-1 expressed GS only in the pericentral region, and Arg1 only in the periportal region (Supplementary Fig. 8f, g), indicating that transplanted IL6-iMHs could be specified into GS or Arg1 positive hepatocytes according to the different zones they were located. The repopulated IL6-td-iMHs were isolated at D63 (Fig. 4a). As expected, these cells all express tdTomato (Supplementary Fig. 9a) without Ki67 and Ck19 staining, but were positive for PAS staining and all mature hepatocyte markers (Supplementary Fig. 9b–d). Moreover, these cells could still be induced to proliferate by IL6 (Fig. 4h, i).

We also tested whether the long-term cultured IL6-td-iMHs (P35, cultured in IL6-HCM for >150 days and then in HMM for another 7 days) and IL6-td-iMHs-clone-1 (P20, cultured in IL6-HCM for ~100 days and then in HMM for another 7 days) could also rescue *Fah*^−/−^ mice. All of the *Fah*^−/−^ mice receiving IL6-td-iMHs (P35) and IL6-td-iMHs-clone-1 (P20) survived for 2 months after NTBC withdrawal (Supplementary Fig. 10a). The livers were almost fully occupied by the td-iMHs (Supplementary Fig. 10b, c). The serum levels of AST, ALT, ALP and total bilirubin were also returned to normal after transplantation (Supplementary Fig. 10d). The repopulated IL6-td-iMHs were almost 100% positive for Albumin staining (Supplementary Fig. 10e), and expressed GS in the pericentral region, and Arg1 in the periportal region (Supplementary Fig. 10f, g). Taken together, these results indicate that IL6-iMHs have functions of mature hepatocytes and can fully repopulate the liver of *Fah*^−/−^ mice after NTBC withdrawal and rescue these animals.

### Diploid cells account for the main expansion population of IL6-iHPCs

**Fig. 5:**
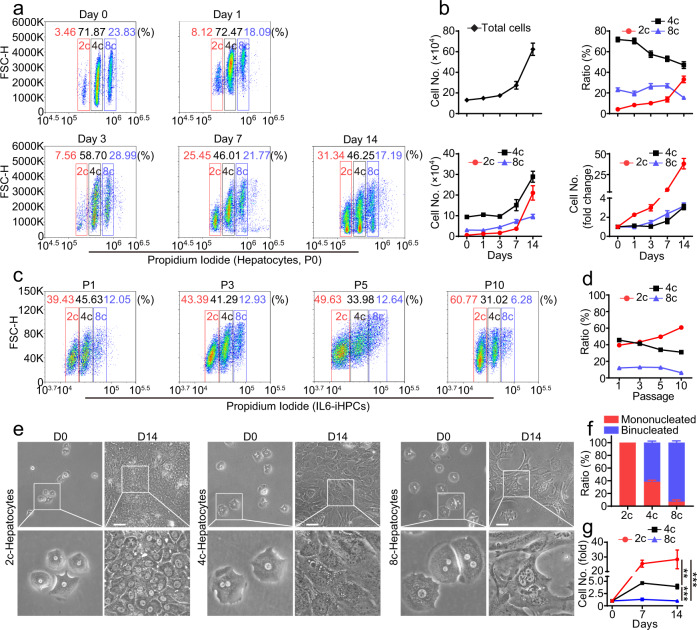
Diploid cells account for the main expansion population of IL6-iHPCs. **a** FACS analysis of ploidy in hepatocytes (P0) cultured in IL6-HCM at day 0, 1, 3, 7 and 14. **b** Statistical data of the total cell number, the ratio of 2c, 4c and 8c cells, the number of 2c, 4c and 8c cells, and fold change of 2c, 4c and 8c cell numbers in **a**. Data are Means ± SEM (*n* = 7 independent experiments). **c** FACS analysis of ploidy in IL6-iHPCs at passage 1, 3, 5 and 10. **d** Statistical data of the percentages of 2c, 4c and 8c cells in **c**. Data are Means ± SEM (*n* = 3 independent experiments). Representative images (**e**) and ratio (**f**) of mononucleated and binucleated cells in FACS sorted 2c, 4c and 8c hepatocytes. Data are Means ± SEM (*n* = 4 independent experiments). **g** Growth curves of sorted 2c, 4c and 8c hepatocytes cultured in IL6-HCM for 14 days. Data are Means ± SEM (*n* = 3 independent experiments). **p < 0.01, ***p < 0.001 (two-way ANOVA). Scale bars represent 100 μm. Source data and exact *p* values are provided in a Source data file.

Previous studies have shown that mononucleated and binucleated polyploid hepatocytes are found in all mammalian species^36^. It is still unclear the exact functional differences between diploid and polyploid hepatocytes, some studies have shown that these cells may behave differently in metabolism, regeneration, tumorigenesis and in response to damage^37–41^. So, we evaluated the proliferation ability of diploid and polyploid hepatocytes in IL6-HCM. Consist with previous reports^36,42,43^, the percentage of diploid (2c), tetraploid (4c) and octaploid (8c) cells in fresh isolated hepatocytes were 4.20 ± 0.63%, 71.83 ± 2.23% and 23.11 ± 1.81%, respectively (Fig. 5a). Interestingly, after culturing in IL6-HCM for 14 days, although the number of 4c and 8c cells were also increasing, but not as fast as the 2c cells, and the percentage of 2c-hepatocytes increased from ~4% (D0) to ~30% (D14), while the percentage of 4c- and 8c-hepatocytes all decreased (Fig. 5b). The number of 2c-hepatocytes increased for ~40 times, and the number of 4c- and 8c-hepatocytes increased for only ~3 times. After serial passage, the percentage of the 2c-population kept increasing from ~40% (P1) to ~60% (P10), while the 4c-population decreased from ~45% (P1) to ~30% (P10) and the 8c-population dropped from 10% (P1) to 5% (P10) (Fig. 5c, d). These data strongly suggest that 2c-hepatocytes are the dominant population during IL6-induced proliferation. However, the ploidy of hepatocytes may change in different cell division stage and may reduce or increase via multipolar mitosis^43^. To better evaluate the proliferation ability of 2c, 4c and 8c-hepatocytes, we sorted these cells with FACS and then cultured them separately in IL6-HCM (Supplementary Fig. 11). Microscopic observations revealed that nearly all 2c-hepatocytes were mononucleated, and only ~40% of 4c-hepatocytes and ~7% of 8c-hepatocytes were mononucleated, and rest of the cells were binucleated (Fig. 5e, f). After culturing in IL6-HCM for 14 days, 2c-hepatocytes (IL6-2c-iHPCs) exhibited the highest expanding ability, the cell number increased for ~30 times (Fig. 5g). And the number of 4c-hepatocytes (IL6-4c-iHPCs) increased ~3 times while no obvious proliferation was observed in 8c cells (Fig. 5g). We also assessed the karyotypes at late passages of IL6-td-iHPCs (P46) and IL6-td-iHPCs-clone-1 (P20). These cells exhibited normal diploid karyotype (Supplementary Fig. 12a, b). In summary, 2c-hepatocytes account for the main expansion population in in vitro culture with IL6-HCM.

### Mechanisms underlying IL6-induced hepatocyte expansion

**Fig. 6:**
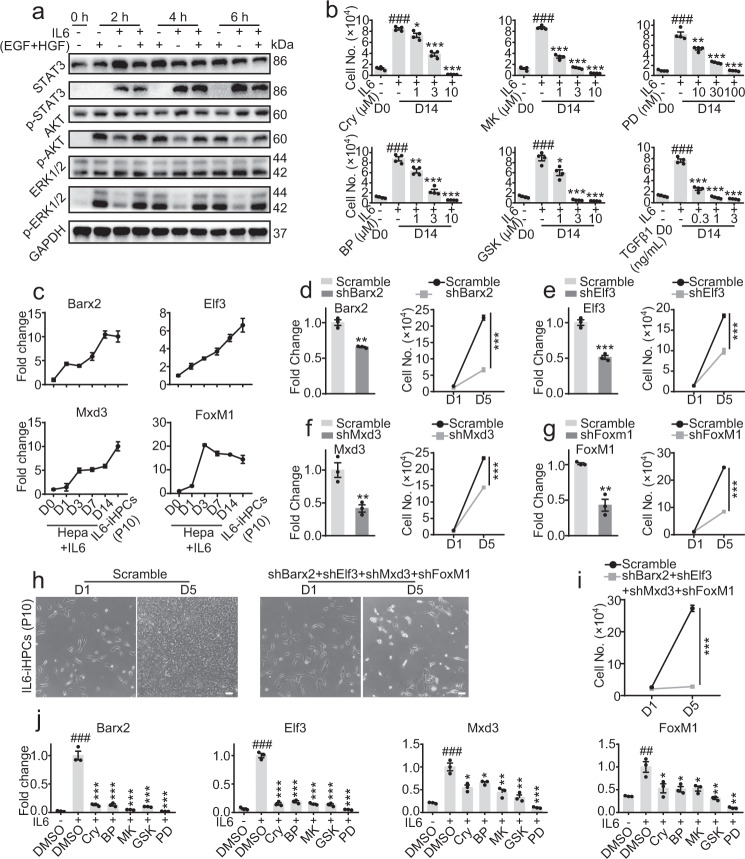
Signal pathways and TFs involved in IL6-induced hepatocyte expansion. **a** Western blot analysis of STAT3, p-STAT3, AKT, p-AKT, ERK1/2, p-ERK1/2 in hepatocytes treated with IL6, EGF/HGF or the combination for 2, 4 and 6 h. GAPDH was used as loading control. **b** Cell numbers of hepatocytes cultured in IL6-HCM and a number of pathway modulators, including Crytotanshinone and BP-1-102 (JAK/STAT3 inhibitor), MK2206 and GSK2141795 (PI3K/AKT inhibitor), PD0325901 (MARK/ERK1/2 inhibitor), and TGFβ1 for 14 days. Data are Means ± SEM (*n* = 4 independent experiments). ^###^*p* < 0.001 vs. first bar, *p < 0.05, **p < 0.01, ***p < 0.001 vs. second bar (two-tailed unpaired Student’s *t* test). **c** Quantitative RT-PCR analysis of *Barx2, Elf3, Mxd3 and FoxM1* in hepatocytes cultured in IL6-HCM for 14 days, and IL6-iHPCs (P10). Data are Means ± SEM (*n* = 3 independent experiments). **d**–**g** Quantitative RT-PCR analysis (left) of *Barx2, Elf3, Mxd3 and FoxM1* expression and cell number (right) of IL6-iHPCs (P10) 5 days after transfection of sh*Barx2*, sh*Elf3*, sh*Mxd3*, sh*FoxM1* or scramble sequence. Data are Means ± SEM (*n* = 3 independent experiments). **p < 0.01, ***p < 0.001 (two-tailed unpaired Student’s *t* test). Representative images (**h**) and cell number (**i**) of IL6-iHPCs (P10) with combination knockdown of *Barx2, Elf3, Mxd3 and FoxM1* in IL6-HCM. Data are Means ± SEM (*n* = 3 independent experiments). **p < 0.01, ***p < 0.001 (two-tailed unpaired Student’s *t* test). **j** Quantitative RT-PCR analysis of *Barx2, Elf3, Mxd3* and *FoxM1* expression in IL6-iHPCs (P10) cultured in IL6-HCM with various pathway inhibitors (Cry, BP, MK, GSK and PD) for 5 days. Data are Means ± SEM (*n* = 3 independent experiments). ^##^*p* < 0.01, ^###^*p* < 0.001 vs. first bar, *p < 0.05, **p < 0.01, ***p < 0.001 vs. second bar (two-tailed unpaired Student’s *t* test). Scale bars represent 100 µm. Source data and exact *p* values are provided in a Source data file.

We have demonstrated in Supplementary Fig. 2e, IL6 in combination with either one of EGF or HGF was enough to convert primary hepatocytes into iHPCs for long-term expansion in vitro. But since EGF + HGF yielded best result and are commonly used in HCM, we’ll consider them as a whole in following study. A number of pathways, including JAK/STAT3, MAPK/ERK and PI3K/AKT have been reported to be downstream of IL6, EGF and HGF^20,25^. Western blotting revealed that during short term treatment (within 6 h), IL6 strongly activated STAT3 and AKT, and slightly activated ERK1/2 pathway; while EGF + HGF strongly activated AKT and ERK1/2, but did not affect STAT3 pathway in hepatocytes (Fig. 6a). During hepatocytes cultured for 14 days in IL6-HCM (containing EGF + HGF), the STAT3 remained highly activated, while AKT was highly activated before Day 1 and maintained at a low activation level. Although the p-ERK1/2 remained at a high level at Day 14, but since total ERK1/2 increased along the time, the percent of activated ERK1/2 actually decreased (Supplementary Fig. 13a). Gene set enrichment analysis (GSEA) also demonstrated that there was a clear enrichment of genes related to IL6-JAK-STAT3 signaling in IL6-iHPCs (Day 14) comparing to primary hepatocytes (Day 0) (Supplementary Fig. 13b). Several pathway inhibitors, including JAK/STAT3 inhibitors Crytotanshinone (Cry) and BP-1-102 (BP), PI3K/AKT inhibitors MK2206 (MK) and GSK2141795 (GSK), and MAPK/ERK1/2 inhibitor PD0325901 (PD), were all found to inhibit IL6 induced hepatocyte proliferation (Fig. 6b), indicating the combined activation of these pathways were necessary. TGFβ, an anti-proliferative factor in liver^20^, also blocked the proliferation (Fig. 6b).

Next, we sought to explore downstream transcription factors involved in IL6 induced hepatocyte proliferation. First line of evidence came from in vivo. After partial PHX, liver regeneration is impaired in the livers of IL6^−/−^ mice^21^, indicating an important role of IL6 in liver regeneration. So, we evaluated the change of transcription factors in livers after PHX (PHX-6 h, 24 h, D3, D7 and D14). Comparing to normal liver, 115 transcription factors which were dramatically increased in PHX-6 h, 24 h or D3 and decreased to normal levels in PHX-D7 and D14 were picked out (Supplementary Fig. 14a, d, Group 1). On the other hand, comparing to primary hepatocytes, 361 transcription factors which were significantly increased in IL6-iHPCs-D3, IL6-iHPCs-D14, IL6-iHPCs-mouse-1 (P10) and IL6-iHPCs-mouse-2 (P10) were identified (Supplementary Fig. 14b, d, Group 2). Moreover, we also compared the transcription factors in IL6-iHPCs-D7 and hepatocytes cultured in HCM (no IL6) for 7 days (HCM-Hepa-D7). Comparing to hepatocytes and HCM-Hepa-D7, 85 transcription factors which were dramatically increased in IL6-iHPCs-D7 were picked out (Supplementary Fig. 14c, d, Group 3). At last, we compared the transcription factors in Group 1, 2 and 3, and 16 genes were identified (Supplementary Fig. 14d). The top 4 genes, including BARX Homeobox 2 (Barx2), E74-like factor 3 (Elf3), Max dimerization protein 3 (Mxd3) and Forkhead box M1 (FoxM1) were evaluated further (Supplementary Fig. 14e–g). Interestingly, FoxM1 was recently reported to promote liver regeneration through a vagus-macrophage-hepatocyte link^44^. Quantitative RT-PCR analysis shown that *Barx2, Elf3, Mxd3* and *FoxM1* in hepatocytes were dramatically increased after cultured with IL6-HCM for 14 days and in IL6-iHPCs (P10) (Fig. 6c). Knockdown of *Barx2, Elf3, Mxd3* or *FoxM1* in IL6-iHPCs (P10) using shRNA dramatically inhibited the growth (Fig. 6d–g). Knockdown of *Barx2, Elf3, Mxd3* and *FoxM1* simultaneously, the IL6-iHPCs (P10) almost lost the growth capacity (Fig. 6h, i). Furthermore, in IL6-iHPCs cultured with pathway inhibitors Cry, BP, GSK, MK or PD for 5 days, *Barx2, Elf3, Mxd3* and *FoxM1* were all dramatically decreased (Fig. 6j), indicating that *Barx2, Elf3, Mxd3* and *FoxM1* were the common downstream of JAK/STAT3, MARK/ERK1/2 and PI3K/AKT pathways. In conclusion, the JAK/STAT3, MAPK/ERK1/2 and PI3K/AKT pathways downstream of IL6, EGF and HGF all contribute to the reprogram of hepatocytes into iHPCs and subsequent proliferation, and these pathways may regulate the growth of iHPCs via upregulation of transcription factors including *Barx2, Elf3, Mxd3* and *FoxM1*.

## Discussion

Adult hepatocytes have amazing proliferative capacity in liver but are very difficult to culture or expand in vitro. Here, we discovered that IL6, in combination with EGF and HGF, can support long-term (>30 passages in ~150 days with theoretical expansion of ~10^35^ times) expansion of hepatocytes in vitro in an easy 2D format by converting the hepatocytes into a progenitor state. IL6 also supports the establishment of single hepatocyte-derived iHPC clones. These IL6-iHPCs can be easily differentiated into mature hepatocytes, which can rescue *Fah*^−/−^ mice after transplantation. This is a culture media very close to physiological conditions. IL6 at 0.1 ng/ml is enough to induce the growth of primary hepatocytes, while 10–30 ng/ml IL6 could support long-term culture. IL6 is typically very low in the serum, less than 1 pg/ml in mice^45^ and less than 100 pg/ml in human^46^. But after hepatectomy, serum IL6 can reach 4 ng/ml in mice^45^ and 0.7 ng/ml in human^46^, and the local concentration of IL6 in the liver might be even higher, since liver macrophages, including Kupffer cells, are the major source of IL6^47^.

TNFα is another inflammatory cytokine that has been found to play a critical role in liver regeneration^20^. TNFα receptor knockout (TNFR-KO) or blocking TNFα with antibodies all impaired liver regeneration in mice or rat^48,49^. However, we found TNFα has limited potential in promoting hepatocyte growth in vitro. Akerman et al. have reported a potential role for TNFα in liver regeneration, they showed that TNFα blockage inhibited the increase in circulating levels of IL6 after PHX^48^. Four hours after PHX, the IL6 mRNA decreased by more than ten-fold in the liver of TNFR-KO mice comparing to wild-mice^49^, and thus the increases in the binding of NFκΒ and STAT3 to DNA also failed to occur^49^. Moreover, the reduced hepatocyte replication and STAT3 binding in TNFR-KO mice could be completely correct by injection of IL6^49^. These results demonstrated a sequence of events for the initiation of liver regeneration: TNFα-NFκB-IL6-STAT3-DNA synthesis-regeneration^49^. TNFα has been reported to regulate function and cytokine secretion of macrophages^49–51^. TNFα has also been found to activate NFκB pathway and IL6 production in Kupffer cells, which leads to STAT3 activation in hepatocytes^52^. Combining these results with our observations, it is clear that TNFα promotes liver regeneration by enhancing IL6 production from macrophages and Kupffer cells, which in turn stimulate hepatocytes proliferation. This may explain why TNFα could induce hepatocytes proliferation in vivo but not in vitro.

We found in hepatocytes, IL6 mainly activates STAT3 pathway, while EGF/HGF mainly contribute to the activation of ERK and AKT pathways. Blocking any one of these pathways leads to failure of hepatocyte expansion, indicating a summation of these pathways is required. Here we identified a group of transcription factors regulated by the combination of STAT3, ERK and AKT pathways, downstream of IL6, EGF and HGF. The top ones being *Barx2, Elf3, Mxd3* and *FoxM1*. Knockdown any of the four reduced the growth the IL6-iHPCs, while simultaneous knockdown of these genes totally blocked proliferation of iHPCs in IL6-HCM. Interestingly, a recent study has reported that *FoxM1* activation is involved in post-injury liver regeneration stimulated by the Vagus-macrophage-hepatocyte network^44^. Compared to *FoxM1*, little is known about the other three TFs in hepatocytes proliferation or liver regeneration. Among them, *Barx2*, a member of the Bar class homeobox gene family, regulates transcription of specific cell adhesion molecules in the mouse^53^. *Barx2* has been reported to regulate muscle growth and repair by regulating satellite cell proliferation and differentiation^54^. *Barx2* has also been reported to function as tumor suppressor in ovarian cancer^53,55^. *Elf3*, a member of the E-twenty-six family of transcription factors, has previously been implicated in the epithelial-mesenchymal transition in hepatocellular carcinoma cells^56^. Upregulation of *Elf3* has been found in NASH induced liver injury, and *Elf3* has also been found to control the genes associating NASH induced reprogramming of hepatocytes to biliary-like hepatocytes^57^. The role of *Mxd3* on liver regeneration was rarely studied, but a study reports that *Mxd3*, as a member of Mad proteins family is highly expressed in fetal liver and low in adult liver^58^, suggesting a possible role of *Mxd3* in hepatocyte proliferation. The exact roles of these genes in in vitro hepatocyte proliferation and in vivo liver regeneration need to be further studies.

Another interesting finding in our study is that the 2c-hepatocytes compose the major expanding population in the in vitro culture system. In adult liver, ~90% of rodent and 50% of human hepatocytes are polyploid^36,59^. Polyploid hepatocytes have been reported to have enhanced functions including metabolism, protein synthesis and secretion^60–62^. In liver regeneration, both diploid and polyploid hepatocytes contribute to liver regeneration^36,38^.However, it has been reported that diploid hepatocytes showed a proliferative advantage, entering and progressing through the cell cycle faster than polyploid cells, both in vitro and during liver regeneration after PHX^43^. On the other hand, in various liver injury models, polyploid hepatocytes have also been found to reduce their ploidy during regeneration^38^. Single-cell analysis has shown that a subpopulation within the 2c-hepatocytes co-expresses the mature hepatocyte markers and HPC markers^42^. Diploid hepatocytes have also been reported to exhibit a stronger binding of insulin than polyploid cells, which is another important factor in liver regeneration^37^. These studies indicate that both the diploid and polyploid hepatocytes can proliferate during liver regeneration. However, in IL6-induced hepatocyte growth in vitro, 2c cells seem to be the only population that can proliferate in long-term culture. After culturing in IL6-HCM for 14 days, the number of FACS-sorted 2c-cells increased for ~30 times (Fig. 5g). The number of FACS-sorted 4c cells only increased ~3 times, and the growth may come from the mixed small amount of 2c cells, which were at G2 stage and inseparable from 4c cells. No proliferation was observed in 8c cells (Fig. 5g), further indicating that 4c cells may not proliferate, since the 8c cells should also contain small amount of 4c cells at G2 stages. More careful studies need to be carried out to fully elucidate the differences observed in in vivo and in vitro hepatocyte growth.

In conclusion, IL6 in combination with EGF and HGF can reprogram primary hepatocytes to iHPCs for long-term expansion without losing the maturation capacity in vitro. IL6-iMHs resemble primary hepatocytes in both gene expression profiles and functions, and can repopulate almost the whole liver of *Fah*^−/−^ mice and rescue them. This physiological and simple way to expand primary hepatocytes in vitro may provide new ideas in culturing previously difficult-to-culture cell types and support their application in regenerative medicine.

## Methods

All the experiments were complied with international guidelines and were approved by the Ethics Committee of Shanghai Institute of Materia Media.

### Mice

All mice were housed under controlled humidity (60 ± 10%) and temperature (22 ± 1 °C) conditions and under 12-h light/dark cycles. The care and the use of animals were complied with international guidelines and were approved by the Animal Ethics Committee of Shanghai Institute of Materia Media.

### Isolation of mice primary hepatocytes

Mice (C57BL/6J) at the age of 8–10 weeks were used. Mouse hepatocytes were isolated using a standard two-step perfusion technique. In brief, the liver was perfused with 15 ml of perfusion buffer (NaCl 8000 mg/l, KCl 400 mg/l, NaH_2_PO_4_ 600 mg/l, Na_2_HPO_4_ 600 mg/l, NaHCO_3_ 350 mg/l, EGTA 190 mg/l, Glucose 900 mg/l, pH 7.35–7.4) through the inferior vena at a flow rate of 3 ml/min, followed by 25 ml Enzyme buffer (NaCl 8000 mg/l, KCl 400 mg/l, NaH_2_PO_4_ 600 mg/l, Na_2_HPO_4_ 600 mg/l, NaHCO_3_ 350 mg/l, CaCl_2_·2H_2_O 560 mg/l, HEPES 190 mg/l, Collagenase Type 1 (Gibco) 600 mg/l, pH 7.35–7.4) at the same flow rate. After perfusion, the liver was removed from the abdominal cavity and hepatocytes were released into the M199 medium supplemented with 10% FBS and 1% penicillin-streptomycin using sterile surgical scissors. Cell suspension was filtered through a 70 μm cell strainer (Corning). After centrifugation, hepatocytes were purified by 50% of percoll (Sigma) gradient at low-speed centrifugation (400 × *g*, 15 min) then the pellets were dissociated into single-cell suspension. Viability of isolated hepatocytes were about 90% as determined by Trypan blue staining. Finally, hepatocytes were seeded into gelatin-coated well plates at a density of 5 × 10^4^/ml. Albumin-Cre mice (Jackson Laboratory, strain number: 003574) were mated with R26RtdTomato mice (Jackson Laboratory, strain number: 007914) to generate mice with specific expression of tdTomato in hepatocytes. The offspring mice at the age of 8–10 weeks were used for lineage tracing experiments (Fig. 3a).

### Generation and culture of IL6-iHPCs

After the primary hepatocytes adhered in 24-well plates for about 6 h, the medium was changed into IL6 expansion medium (IL6-HCM: DMEM/F12, 1 × ITS (Sigma), 20 ng/ml EGF (Sigma), 20 ng/ml HGF (Gibco), 10^−7^ M Dexamethasone (Sigma), 30 ng/ml IL6 (R&D Systems) and 1% penicillin-streptomycin. At day 14, the IL6-iHPCs were trypsinized into single cells and washed with DMEM/F12 supplemented with 10% FBS, then the IL6-iHPCs were reseeded into gelatin-coated 6-well plates at a density of 1 × 10^5^/well and cultured in IL6-HCM, the medium was changed every other day.

### Cell differentiation

For induction of hepatic maturation, IL6-iHPCs growth in IL6-HCM until 90% confluence. Then the medium was changed into HMM (DMEM/F12 (Gibco), 1 × ITS (Sigma), 10^−6^ M Dexamethasone (Sigma), 20 ng/ml OSM (Gibco), 1% penicillin-streptomycin, the medium was changed for every 2 days for 7 days.

### Cell counting

To evaluate cell proliferation, cells were fixed with 4% paraformaldehyde (PFA) at room temperature for 30 min and then washed three times with PBS. Then, Nuclei were stained with Hoechst 33342 (10 μg/ml) for 30 min. One field per well was imaged using the ×10 objective on a PerkinElmer Opera Phenix High Content Screening System and the images were analyzed using the associated Harmony Office Software.

### Immunofluorescence staining, PAS staining and ICG assay

Tissue slides were incubated in PBS containing 0.3% Triton X-100 and 5% BSA for 1 h. The slides were incubated with primary antibodies at 4 °C overnight. After thorough wash, slides were incubated with the appropriate fluorescence-conjugated secondary antibodies (1:500) for 1 h at room temperature. Finally, the cell nuclei were stained with Hoechst 33342 solution for 30 min. Cells were fixed in 4% PFA at room temperature for 30 min, then follow the same staining protocol as tissue slides. Images were captured with an Olympus IX71 inverted fluorescent microscope and analyzed by professional image analysis software (ImageJ).

The following antibodies were used in this study: CyclinD1 (1:200; Abcam ab134175), Ki67 (1:200; Cell Signaling Technology 9129S), Sox9 (1:200; Millipore ab5535), Cytokeratin 19 (1:200; Abcam ab52625), Albumin (1:200; Bethyl, A90-234A), Hnf4α (1:200; Abcam ab181604), Cyp1a2 (1:200; Abcam ab22717), Cyp2c9 (1:200; Abcam ab4236), Fah (1:200; GeneTex GTX114400), GS (1:500; Abcam, ab49873), Arg1 (1:200; Abcam, ab96183). Secondary antibodies including Alexa Fluor 555 goat anti-mouse IgG (#A32727), Alexa Fluor 555 goat anti-rabbit IgG (#A32732), Alexa Fluor 488 rabbit anti-goat IgG (#A27012), Alexa Fluor 488 goat anti-mouse IgG (#A32723) and Alexa Fluor 488 goat anti-rabbit IgG (#A11034) were purchased from Invitrogen.

To analyze the glucose storage ability of primary hepatocytes and IL6-iMHs, cells were fixed by 4% PFA and staining with PAS kit (Sigma) according to the manufacturer’s instructions. To analyze the ICG uptake capacity of primary hepatocytes and IL6-iMHs, the HMM was replaced by fresh medium supplied with 1 mg/ml ICG (US Pharmacopeia, 1340009) and incubated for 1 h, then the cell fixed by 4% PFA for 30 min. Images were captured with an Olympus IX71 inverted fluorescent microscope.

### Albumin ELISA, urea assay

To analyze albumin secretion ability, primary hepatocytes and IL-6iMHs were cultured for 24 h, and the supernatants were collected and measured using a mouse serum albumin kit (Abcam, ab108792) according to the manufacturer’s instructions. Mouse urea were detected using a Urea Assay Kit (Sigma-Aldrich, MAK006), according to the corresponding manufacturer’s instructions. The absorbance signals were measured with the EnSight™ multimode plate reader (PerkinElmer).

### Cyp450 metabolism assay

To measure Cyp450 enzyme activity, primary hepatocytes and IL6-iMHs were cultured in HMM containing 100 μM phenacetin, 100 μM diclofenac and 100 μM testosterone for 24 h. Then, the supernatants were collected. Concentrations of the metabolites (acetaminophen, 4’-OH-diclifenac and 6β-OH-testosterone) in the supernatants were measured via liquid chromatograph-tandem mass spectrometer (Waters UPLC I-Class and Waters Xevo TQ-S mass spectrometer). All compounds were also purchased from Sigma-Aldrich and used as standard samples.

### Assessment of anti-IL6 antibody

The primary hepatocytes or IL6-iHPCs (P5) were cultured in IL6-HCM and supplied with different concentrations of control IL6 neutralizing antibody (R&D Systems, MAB406-SP) or IgG (R&D Systems, 6-001-A) as shown in Supplementary Fig. 2a–d, the medium was changed for every other day.

### Assessment of PI3K/AKT, ERK1/2, STAT3 pathways inhibitors and TGFβ1

The primary hepatocytes were cultured in IL6-HCM and supplied with different concentrations of chemicals or TGFβ1 (R&D Systems, 240-B-010) as shown in Fig. 6b. All chemicals were purchased from MedChemExpress, the medium was changed for every other day.

### Establishment of single hepatocytes derived IL6-iHPC clones

The single primary hepatocytes were pipetted under fluorescence microscope and seeded in one well of 96-well plates. After cultured in IL6-HCM for 14 days, the single primary hepatocytes derived IL6-iHPC clones were trypsinized and reseeded into new 12-well plate for further expanding.

### Quantitative RT-PCR analysis

Total mRNA was isolated using Trizol (Invitrogen) and 1 μg RNA was reversed to cDNA using PrimeScriptTM RT reagent kit (Takara) according to the manufacturer’s protocol. Real-time PCR was performed using Hieff Qpcr SYBR Green Master Mix (Yeasen) and analyzed with a Stratagene Mx 3000P thermal cycler. Primers sequences are supplied in Supplementary Table 2.

### Flow cytometry analysis of hepatocytes ploidy populations

Primary hepatocytes and IL6-iHPCs were fixed and permeabilized with Fixation/Permeabilization buffer (eBioscience™, 005123-43) for 20 min at 4 °C, after thorough wash, cells were incubated with 10 μg/ml propidium iodide (containing 0.2 mg/ml RNAase) for 30 min at room temperature, then cells were transferred into the 96-well plate through a 40 μm filter membrane, and then analyzed using a flow cytometer (ACEA Novocyte).

### FACS sorting of 2c, 4c, 8c-hepatocytes

Primary hepatocytes were suspended in DMEM/F12 supplemented with 50 mg/ml Hoechst 33342 and 5 μM reserpine and incubated at 37 °C for 10 min. Following the addition of 5 μg/ml propidium iodide (Yeasen, 40711ES10), the cells were analyzed using a FACS Aria III system (BD) as described in Supplementary Fig. 11 and sorted into 24-well collagen-coated plates for culture.

### Karyotyping analysis

Cultured IL6-iHPCs in exponential growing phase were incubated with IL6-HCM containing 100 ng/ml colcemid for 3–4 h at 37 °C. Then, the cells were harvested via trypsinization and were treated with 0.075 M KCl at 37 °C for 30 min, and then fixed in methanol: acetic acid (3:1 ratio), with the solution changed two times. Finally, chromosomes were spread on slide glasses and stained with Giemsa staining solution (Gibco).

### 2/3 partial hepatectomy (2/3 PHx)

Mice (C57BL/6J) at the age of 8–10 weeks were used for this experiment. 2/3 PHx operation was performed as previously reported^63^. Adult mice were sacrificed at 6 h, 24 h, day 3, day 7 and day 14 to obtain liver tissues.

### Bioinformatic analysis

RNA library for RNA-sequencing was prepared as follows: Firstly, total RNA was isolated using Trizol according to the manufacturer’s instructions. Secondly, mRNA was purified from total RNA and then was reverse-transcribed to cDNA for sequencing. Finally, after library preparation and pooling of different samples, the samples were subjected for Illumina sequencing using PE150 (paired-end 150nt) sequencing. Heatmap and correlation map were drawn by R software (v4.2.1, https://www.r-project.org). Genes with median absolute deviation (MAD > 2) were used for hierarchical clustering. GSEA was performed to identify the significantly enriched pathways of hepatocytes culturing with IL6 for 14 days. (Website: gsea-msigdb.org; Software: GSEA v4 desktop).

### Western blot

Cells were lysed and homogenized in RIPA buffer supplemented with a protease inhibitor cocktail (Roche). Total protein was quantified using the BCA Protein Assay Kit (Thermo Fisher). Then protein extracts were boiled at 100 °C for 10 min in sample buffer (50 mM Tris-HCl, 2% w/v SDS, 10% glycerol, 1% β-mercaptoethanol, 0.01% bromophenyl blue (pH 6.8)). Cell lysates were separated on SDS-PAGE and transferred to polyvinylidene difluoride membranes. The membranes were first incubated with blocking buffer (TBS with 0.05% Tween 20, 10% nonfat milk) for 1 h at room temperature and then incubated overnight at 4 °C in buffer containing primary antibody. After thorough wash, cells were incubated with the appropriate HRP-conjugated secondary antibodies (1:10,000) for 1 h. Immunostaining was visualized using Amersham ECL Plus Western Blotting detection reagents (GE Healthcare) and ChemiDoc imaging system (Bio-Rad). The uncropped and unprocessed scans of the blots were provided in Supplementary Fig. 15.

Antibodies used include anti-STAT3 (1:1000; CST 9139S), anti-p-STAT3 (1:1000; CST 9145S); anti-Akt (1:1000; CST 9272S); anti-p-Akt (1:1000; CST 4060S); anti-ERK1/2 (1:1000; CST 4695S); anti-p-ERK1/2 (1:1000; CST 4370S); anti-GAPDH (1:8000; CST 2118S); HRP-linked anti-rabbit IgG (1:10,000; CST 7074S), HRP-linked anti-mouse IgG (1:10,000 CST 7076S).

### Hematoxylin and eosin (H&E) staining

Liver samples were fixed in 4% paraformaldehyde (PFA), embedded in paraffin, and cut into 5 μm-thick sections. For H&E staining, sections were first stained with hematoxylin solution for 10 min, and then stained with eosin alcohol solution for 5 min. Finally, the sections were dehydrated and mounted with Permount Mounting Medium.

### Hepatocyte transplantation and samples collection

All procedures for the care and the use of animals were complied with international guidelines and were approved by the Animal Ethics Committee of Shanghai Institute of Materia Media. *Fah*^−/−^ mice (C57BL/6J background) at the age of 8–10 weeks were used for this experiment. Before transplantation, *Fah*^−/−^ mice were fed with 7.5 mg/l NTBC in drinking water. For cell transplantion, according to previous reports, hepatocytes (2.5 × 10^6^) were suspend in 250 μl 0.9% sodium chloride solution and transplanted into *Fah*^−/−^ mice via intrasplenic injection through a left-flank incision under 1.25% tribromoethanol anesthesia. After the operation, NTBC was withdrawn from the drinking water immediately. The blood and liver samples of *Fah*^−/−^ mice transplanted with vehicle were collected at day 28. The blood and liver samples of *Fah*^−/−^ mice transplanted with hepatocytes were collected at day 63. ALT, AST, ALP and TBIL were determined by commercial kits (BioAssay Systems) and a multimode plate reader.

### Barx2, Elf3, FoxM1 and Mxd3 knockdown in IL6-iHPCs (P10)

Short hairpin RNA (shRNA) targeting mouse *Barx2*, *Elf3*, *FoxM1* and *Mxd3* mRNA (Supplementary Table 3) were cloned into the PLKO.1/U6 plasmid. HEK293T cells were co-transfected with PLKO.1 (lentiviral plasmid), psPAX2 (packaging plasmid) and pMD2.G (envelope plasmid). Supernatants were collected 72 h after transfection. Lentiviral particles were filtered and stored at −80 °C until use. IL6-iHPCs (P10) were seeded in 24-well plates at a density of 2 × 10^4^/well. Then the medium was replaced by virus-containing supernatant supplemented with 8 μg/ml polybrene (Millipore), and the plates were centrifuged at 1000 × *g* for 90 min to ensure viral infection, after infection the medium was changed to IL6-HCM for further culture. Knockdown efficiency was determined using qRT-PCR and cell counting later.

### Statistical analysis

Values are reported as means ± SEM. *p* values were calculated by Student’s *t* test for two groups. To compare the curves for two groups, two-way ANOVA was performed in Figs. 1c, 4b, i and 5g. Survival was analyzed with Log-rank test in Fig. 4c and Supplementary Fig. 10a. *p* values <0.05 were considered statistically significant. All graphs were plotted with GraphPad Prism software (version 8.0.1). The Immunofluorescence staining and positive cell percentage were analyzed using ImageJ software (version 1.46r).

### Reporting summary

Further information on research design is available in the Nature Portfolio Reporting Summary linked to this article.

## Supplementary information

Supplementary Information

Reporting Summary

## Data Availability

The microarray data are available at the NCBI Gene Expression Omnibus with accession number GSE215423. Source data are provided with this paper.

## Source data

Source Data
