# Supplementary material for: IL6 supports long-term expansion of hepatocytes in vitro
Source: Nat Commun. 2022 Nov 29;13:7345. doi: 10.1038/s41467-022-35167-8 (PMC9708838; doi:10.1038/s41467-022-35167-8)
Supplement: Supplementary file 1 — Supplementary Information [file 41467_2022_35167_MOESM1_ESM.pdf]

Supplementary Information

IL6 Supports Long-Term Expansion of Hepatocytes *in vitro*

Ren Guo[1,8], Mengmeng Jiang[1,2,3,8], Gang Wang[4,8], Bing Li[1,3,5,8], Xiaohui Jia[1,3], Yan Ai[1,3,5], Shanshan Chen[1,3], Peilan Tang[1,7], Aijie Liu[1], Qianting Yuan[1], Xin Xie[1,2,3,4,5,6,*]

[1]State Key Laboratory of Drug Research, National Center for Drug Screening, Shanghai Institute of Materia Medica, Chinese Academy of Sciences, Shanghai 201203, China

[2]School of Life Science and Technology, Shanghai Tech University, Shanghai 201210, China

[3]University of Chinese Academy of Sciences, No.19A Yuquan Road, Beijing 100049, China

[4]Department of Pharmaceutics, School of Pharmacy, Fudan University, Shanghai 201203, China

[5]School of Pharmaceutical Science and Technology, Hangzhou Institute for Advanced Study, University of Chinese Academy of Sciences, Hangzhou, 310024, China

[6]Shandong Laboratory of Yantai Drug Discovery, Bohai Rim Advanced Research Institute for Drug Discovery, Yantai, Shandong 264117, China

[7]School of Pharmaceutical Science, Nanchang University, Nanchang 330006, China

[8]These authors contributed equally: Ren Guo, Mengmeng Jiang, Gang Wang, Bing Li

*Address correspondence to: Dr. Xin Xie, 189 Guo Shou Jing Road, Shanghai 201203, China; Tel: (86) 186-0211-0377; Fax: 0086-21-50800721; E-mail: xxie@simm.ac.cn.

Running title: IL6 promotes hepatocyte growth *in vitro*

Supplementary Fig. 1. Effects of TNFα and IL6 in hepatocyte proliferation

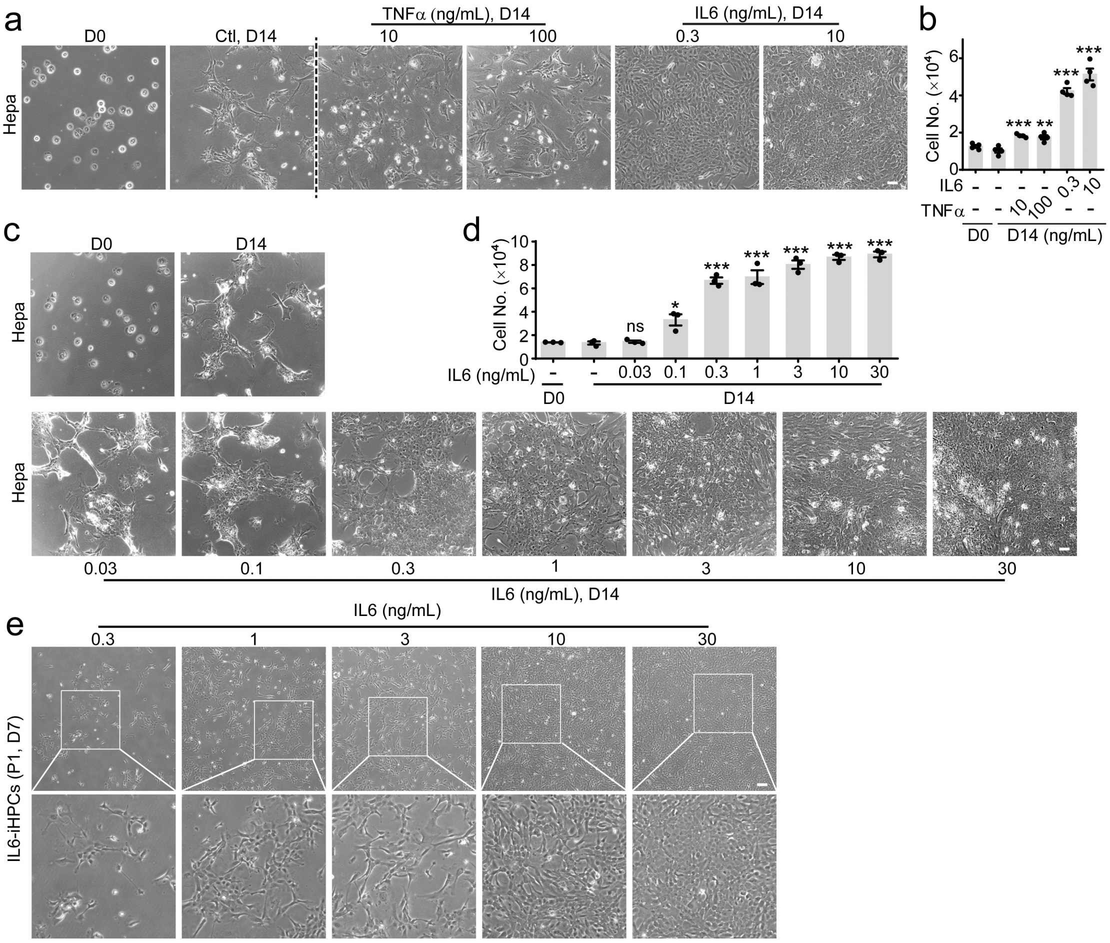

(**a**, **b**) Representative phase contrast images and cell numbers of primary hepatocytes (Hepa) cultured in media supplemented with TNFα (10, 100 ng/mL) or IL6 (0.3, 10 ng/mL) for 14 days (n=4 independent experiments). (**c**, **d**) Representative images (**c**) and cell numbers (**d**) of primary hepatocytes cultured in different concentration of IL6 for 14 days (n=3 independent experiments). (**e**) Representative images of IL6-iHPCs (P1) cultured in different concentration of IL6 for 7 days (repeated for 3 times). All data are shown as Means ± SEM. ns, not significant, *P < 0.05, **P< 0.01, ***P< 0.001 versus ctl group in D14 (two-tailed unpaired Student's t test). Source data and exact P values are provided in a Source data file. Scale bars represent 100 μm.

Supplementary Fig. 2 IL6 promotes primary hepatocytes proliferation *in vitro*

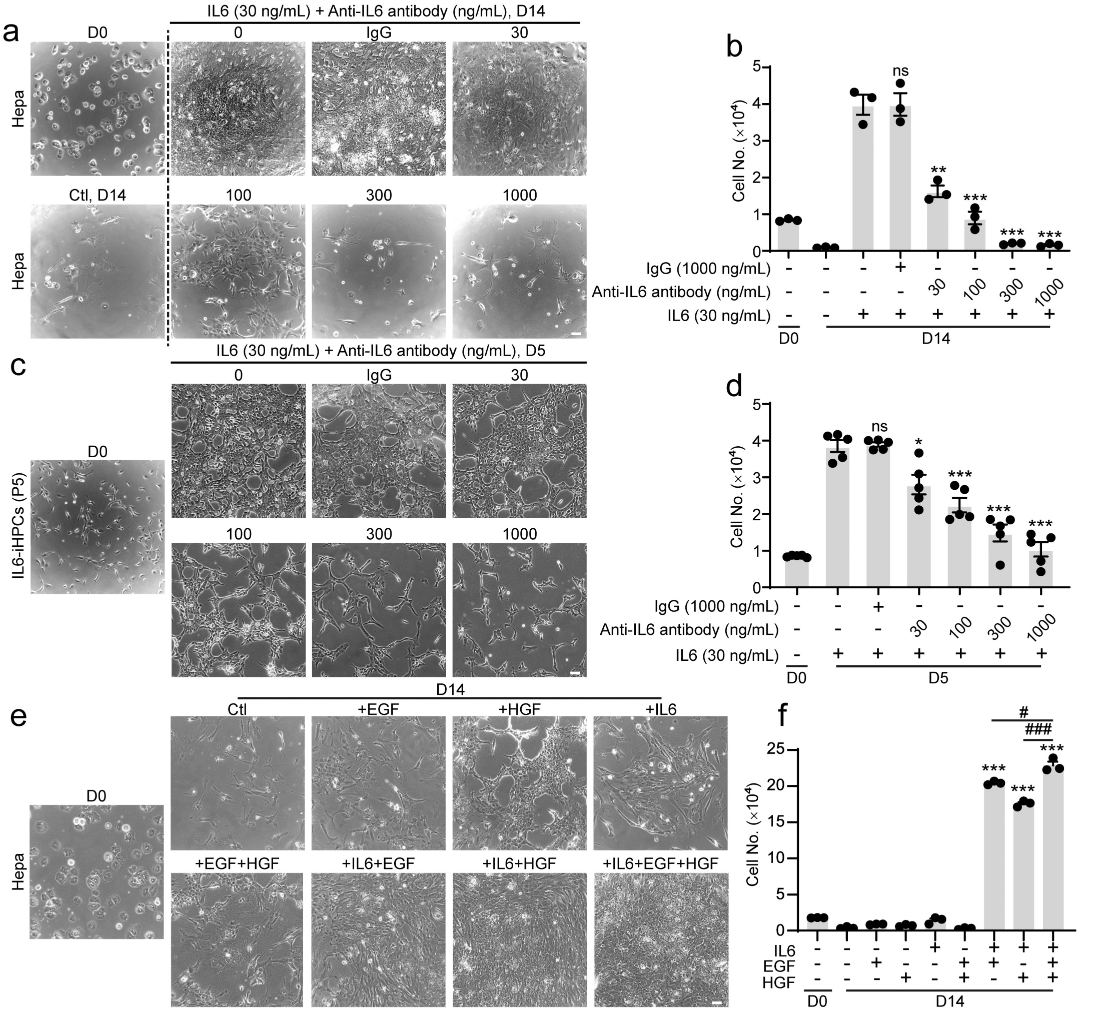

(**a**, **b**) Representative images (**a**) and cell numbers (**b**) of primary hepatocytes cultured in IL6 and various concentration of anti-IL6 antibody or control IgG for 14 days (n=3 independent experiments). ns, not significant, **P < 0.01, ***P < 0.001 versus IL6 (30 ng/mL, D14) alone. (**c**, **d**) Representative images (**c**) and cell numbers (**d**) of IL6-iHPCs (P5) cultured in IL6 and various concentration of anti-IL6 antibody or control IgG for 5 days (n=5 independent experiments). ns, not significant, *P < 0.05, ***P< 0.001 versus IL6 (30 ng/mL, D5) alone. (**e**, **f**) Representative images (**e**) and cell numbers (**f**) of primary hepatocytes cultured in EGF (20 ng/mL), HGF (20 ng/mL)，IL6 (30 ng/mL), or various combinations, for 14 days (n=3 independent experiments). ***P< 0.001 versus first bar in D14 group, $^{\#}$P< 0.05, $^{\#\#\#}$P< 0.001. All data are shown as Means ± SEM. P-values were calculated by two-tailed unpaired Student’s t test. Source data and exact P values are provided in a Source data file. Scale bars represent 100 μm.

Supplementary Fig. 3 IL6-iHPCs could be differentiated into mature hepatocytes

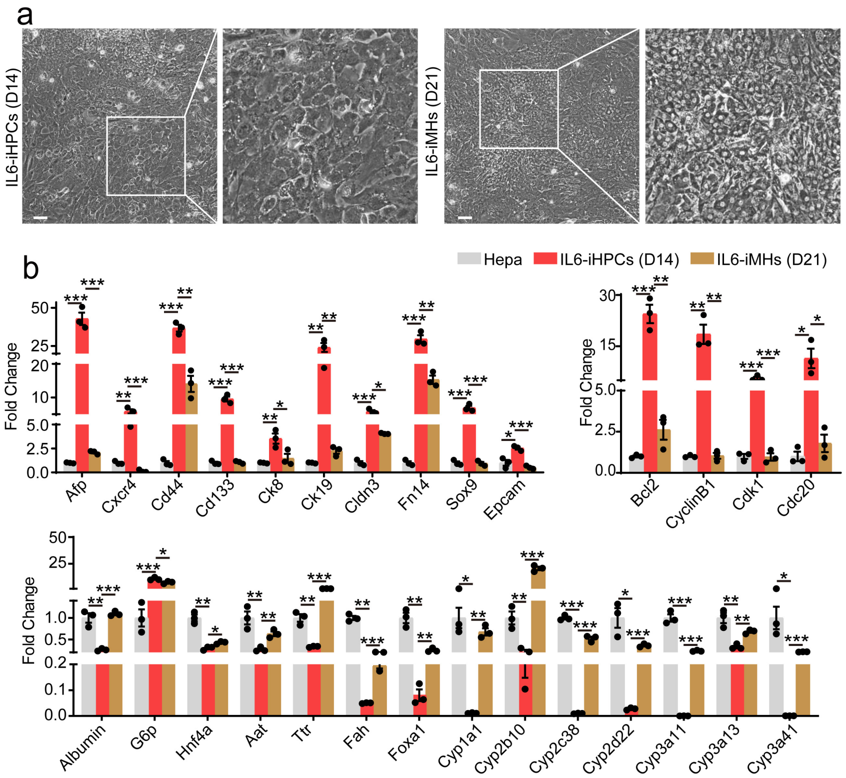

(**a**) Representative images of IL6-iHPCs (D14) and IL6-iMHs (D21). (**b**) Quantitative RT-PCR analysis of hepatic progenitor genes including *Afp, Cxcr4, Cd44, Cd133, Ck8, Ck19, Cldn3, Fn14, Sox9* and *Epcam,* cell cycle genes including *Bcl2, CyclinB1, Cdk1,* and *Cdc20,* and hepatic genes including *Albumin, G6p, Hnf4a, Aat, Ttr, Fah, Foxa1, Cyp1a1, Cyp2b10, Cyp2c38, Cyp2d22, Cyp3a11, Cyp3a13, and Cyp3a41* in primary hepatocytes (Hepa), IL6-iHPCs (D14) and IL6-iMHs (D21) (n=3 independent experiments). All data are shown as Means ± SEM, *P < 0.05, **P< 0.01, ***P< 0.001 (two-tailed unpaired Student's t test). Source data and exact P values are provided in a Source data file. Scale bars represent 100 μm.

Supplementary Fig. 4 Characterization of long-term expanded IL6-iHPCs

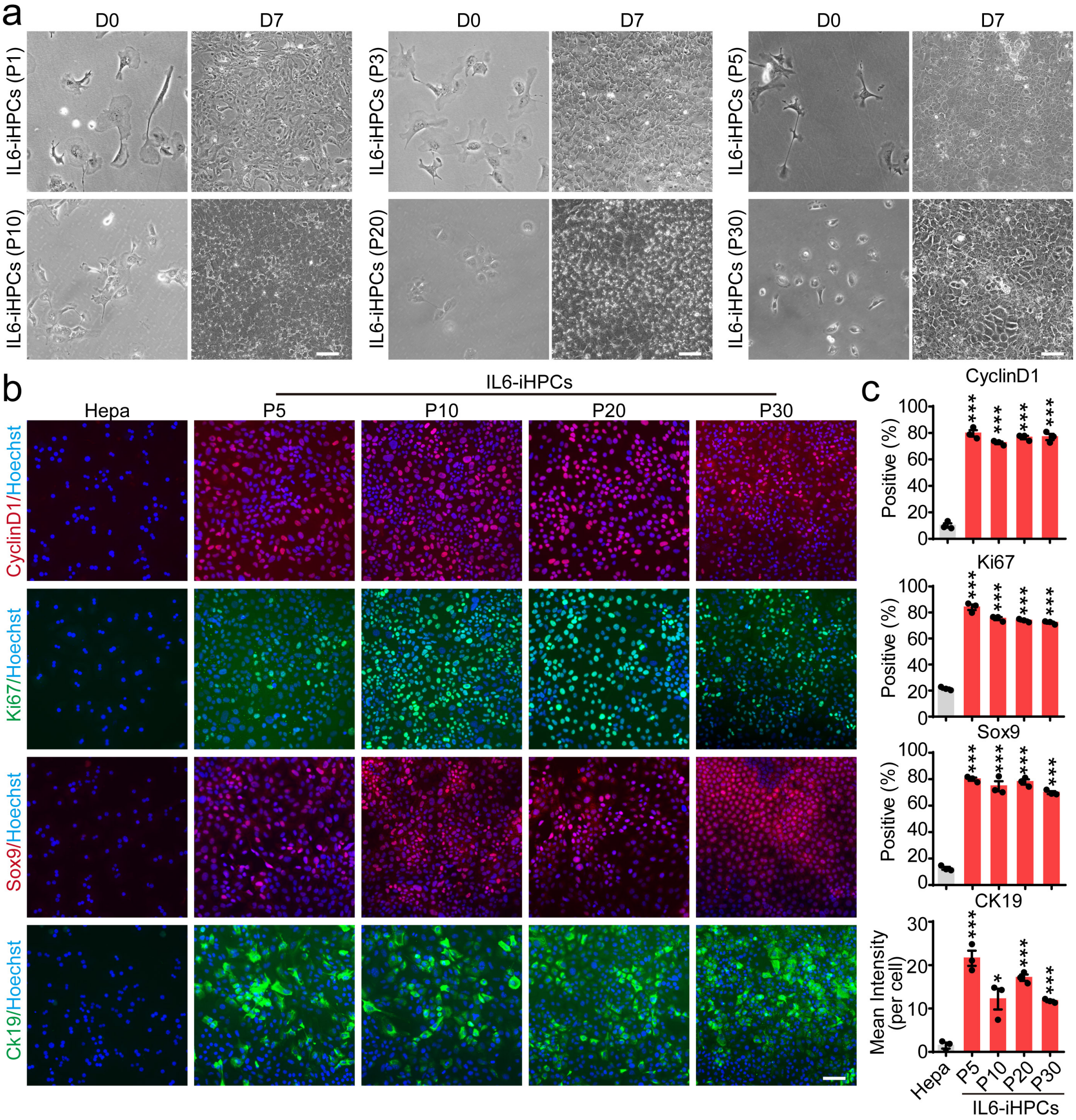

(**a**) Representative images showing the proliferation ability of IL6-iHPCs passaged for 1, 3, 5, 10, 20 and 30 times (repeated for 3 times). For readers' convenience, the images of IL6-iHPCs at P10 and P30 are also presented in Fig. 2b. (**b**, **c**) Immunofluorescence staining (**b**) and statistical data (**c**) of cell cycle markers CyclinD1 (red) and Ki67 (green) and hepatic progenitor cell markers Sox9 (red) and Ck19 (green) in IL6-iHPCs at different passage (n=3 independent experiments). Nuclei were stained with Hoechst 33342 (blue). All data are shown as Means ± SEM, $*P < 0.05$, $***P < 0.001$ versus primary hepatocytes (two-tailed unpaired Student's t test). Source data and exact P values are provided in a Source data file. Scale bars represent 100 μm.

Supplementary Fig. 5 Characterization of IL6-iMHs differentiated from IL6-iHPCs

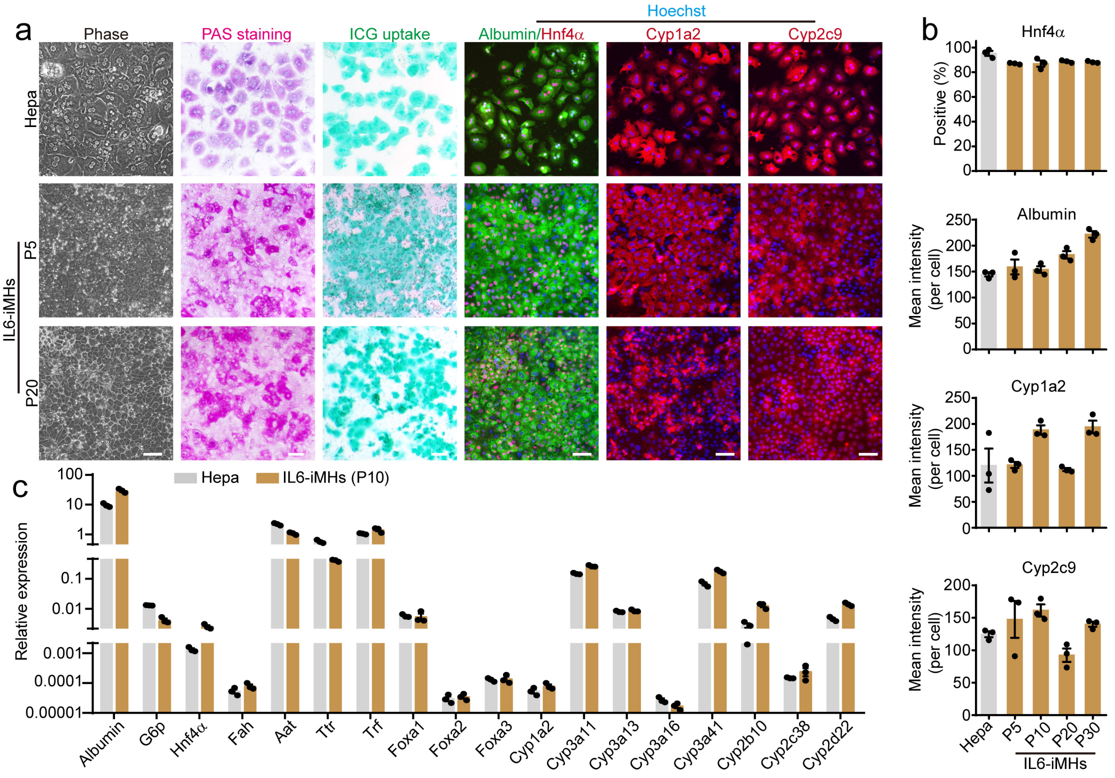

(**a**) Representative phase contrast images, PAS staining, ICG uptake and immunofluorescence staining of hepatic markers Albumin (green), Hnf4α (red), Cyp1a2 (red), and Cyp2c9 (red) in IL6-iMHs (P5 and P20). Primary hepatocytes (Hepa) were used as control. Nuclei were stained with Hoechst 33342 (blue). (**b**) Statistical data of the immunofluorescence staining data in (**a**) and Figure 2D (n=3 independent experiments). (**c**) Quantitative RT-PCR analysis hepatic genes including *Albumin, G6p, Hnf4α, Fah, Aat, Ttr, Trf, Foxa1, Foxa2, Foxa3, Cyp1a2, Cyp3a11, Cyp3a13, Cyp3a16, Cyp3a41, Cyp2b10, Cyp2c38,* and *Cyp2d22* in IL6-iMHs (P10) and Hepa (n=3 independent experiments). All data are shown as Means ± SEM. Source data are provided in a Source data file. Scale bars represent 100 μm.

Supplementary Fig. 6 Characterization of IL6-td-iHPC clones generated from single cells

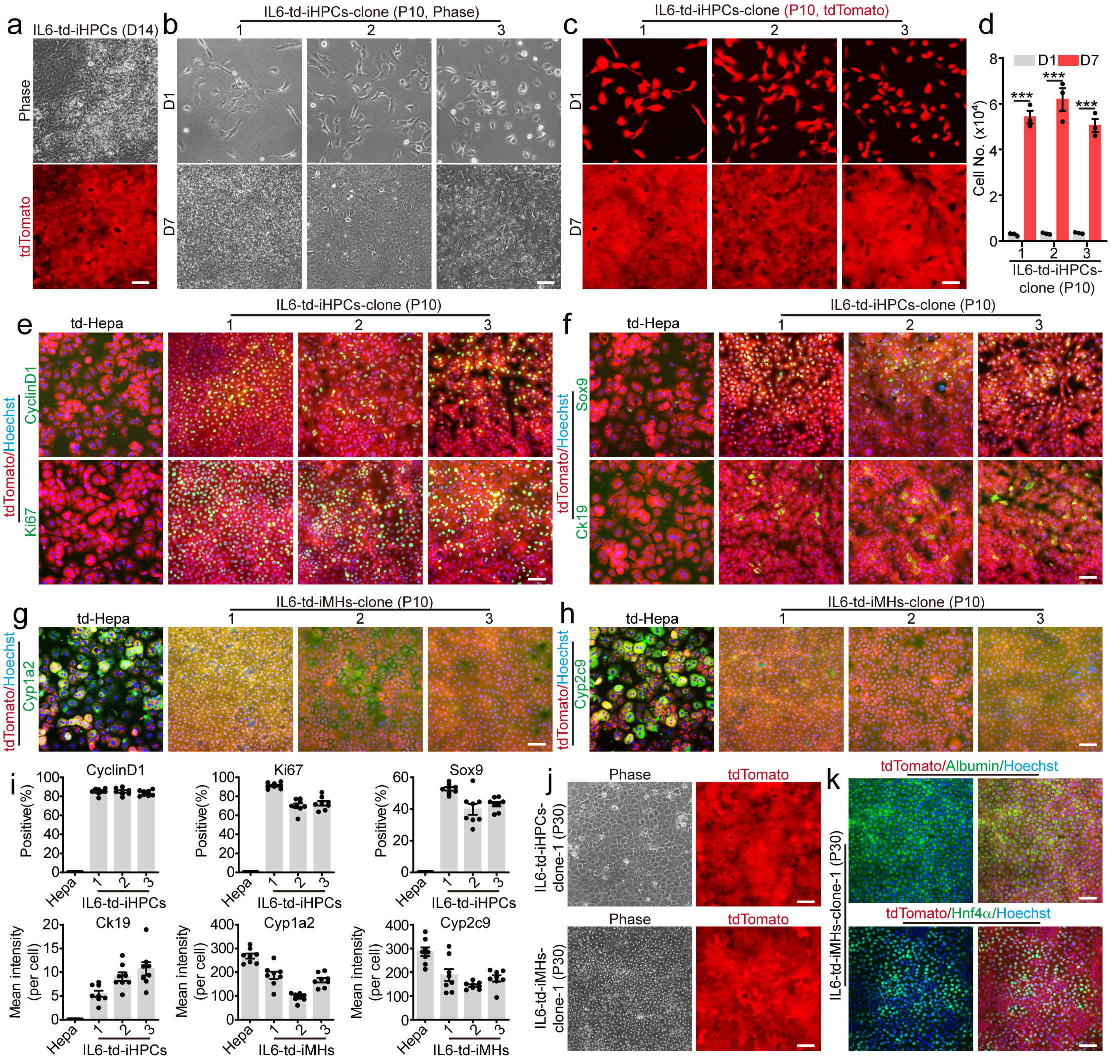

(**a**) Representative morphology and fluorescence images of td-Hepa (red) cultured in IL6-HCM for 14 days. (**b-d**) Representative morphology (**b**), fluorescence images (**c**) and cell numbers (**d**) of 3 single hepatocyte-generated IL6-td-iHPC clones (P10) cultured in IL6-HCM for 7 days (n=3 independent experiments). ***$P < 0.001$ (two-tailed unpaired Student's t test). (**e**, **f**) Immunofluorescence staining of cell cycle markers CyclinD1 (green) and Ki67 (green) (**e**) and hepatic progenitor cell marker Sox9 (green) and Ck19 (green) (**f**) in IL6-td-iHPC clones (P10). (**g**, **h**) Immunofluorescence staining of hepatic markers Cyp1a2 (green) (**g**) and Cyp2c9 (green) (**h**) in IL6-td-iMH clones (P10). Nuclei were stained with Hoechst 33342 (blue). (**i**) The statistical analysis of **e-h** (eight random fields each group, 3 independently generated IL6-td-iMH clones

were analyzed). (**j**) Representative morphology and fluorescence images (red) of IL6-td-iHPC-clone-1 and IL6-td-iMHs-clone-1 (P30). (**k**) Immunofluorescence staining of hepatic markers Albumin (green) and Hnf4α (green) in IL6-td-iMHs-clone-1 (P30). Nuclei were stained with Hoechst 33342 (blue). All data are shown as Means ± SEM. All images are representative images from at least 3 independent experiments. Source data and exact P values are provided in a Source data file. Scale bars represent 100 μm.

Supplementary Fig. 7 Transcriptome analysis of IL6-iHPCs and IL6-iMHs

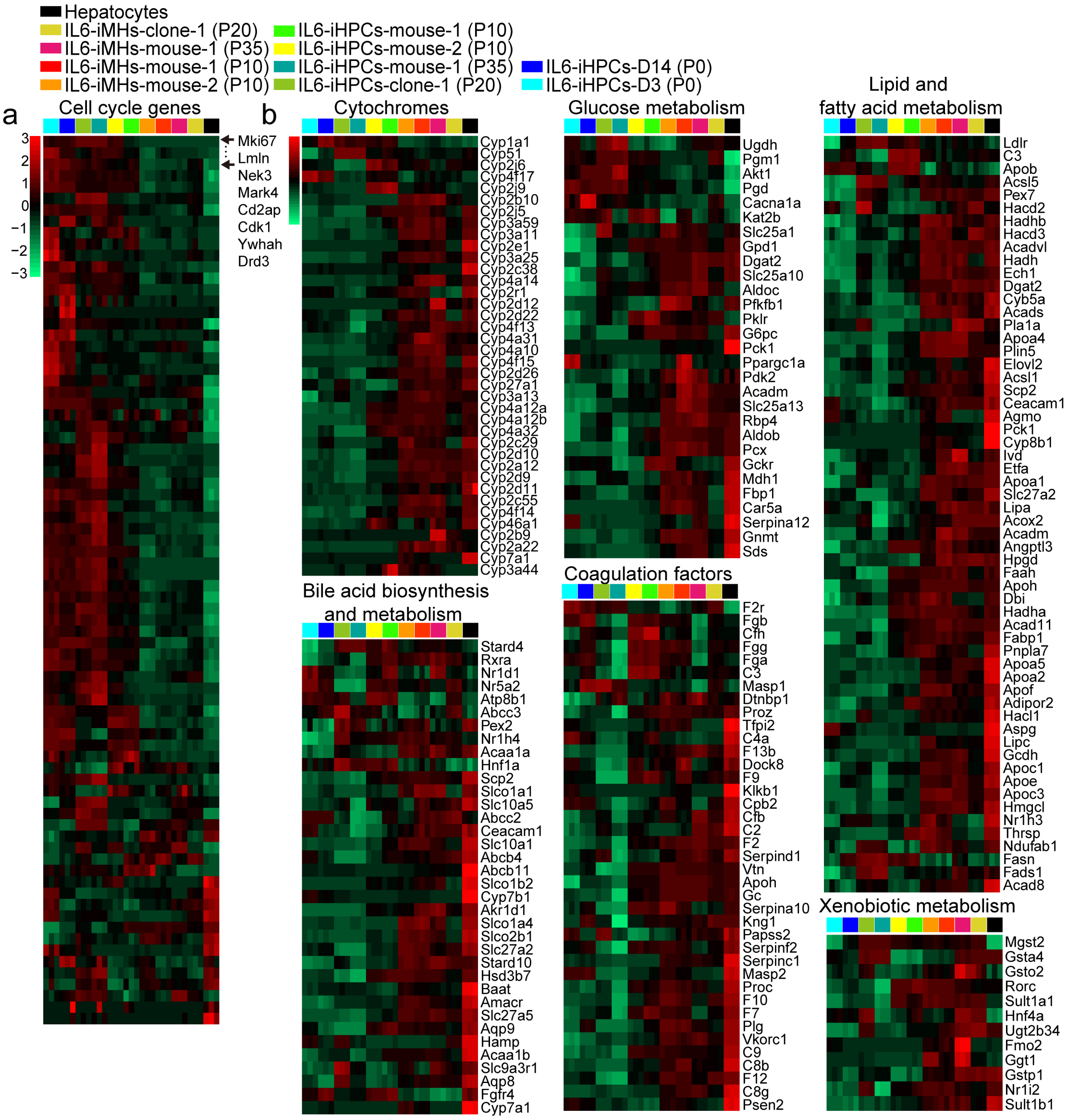

(**a**, **b**) Heatmaps for the expression of cell cycle genes (**a**) and representative hepatic function-related genes: Cytochromes, Bile acid biosynthesis and metabolism, Glucose metabolism, Coagulation factors, Lipid and fatty acid metabolism and Xenobiotic metabolism (**b**) in primary hepatocytes, IL6-iHPCs-D3 (P0), IL6-iHPCs-D14 (P0), IL6-iHPCs from different mouse and different passages and the IL6-iMHs differentated from them. Gene expression is presented in log 2 scale as defined by the corresponding color bars in the left. Red and green represent higher and lower gene expression levels respectively. Three technical replicates for all groups.

Supplementary Fig. 8 Repopulation of *Fah*$^{-/-}$ liver by IL6-iMHs

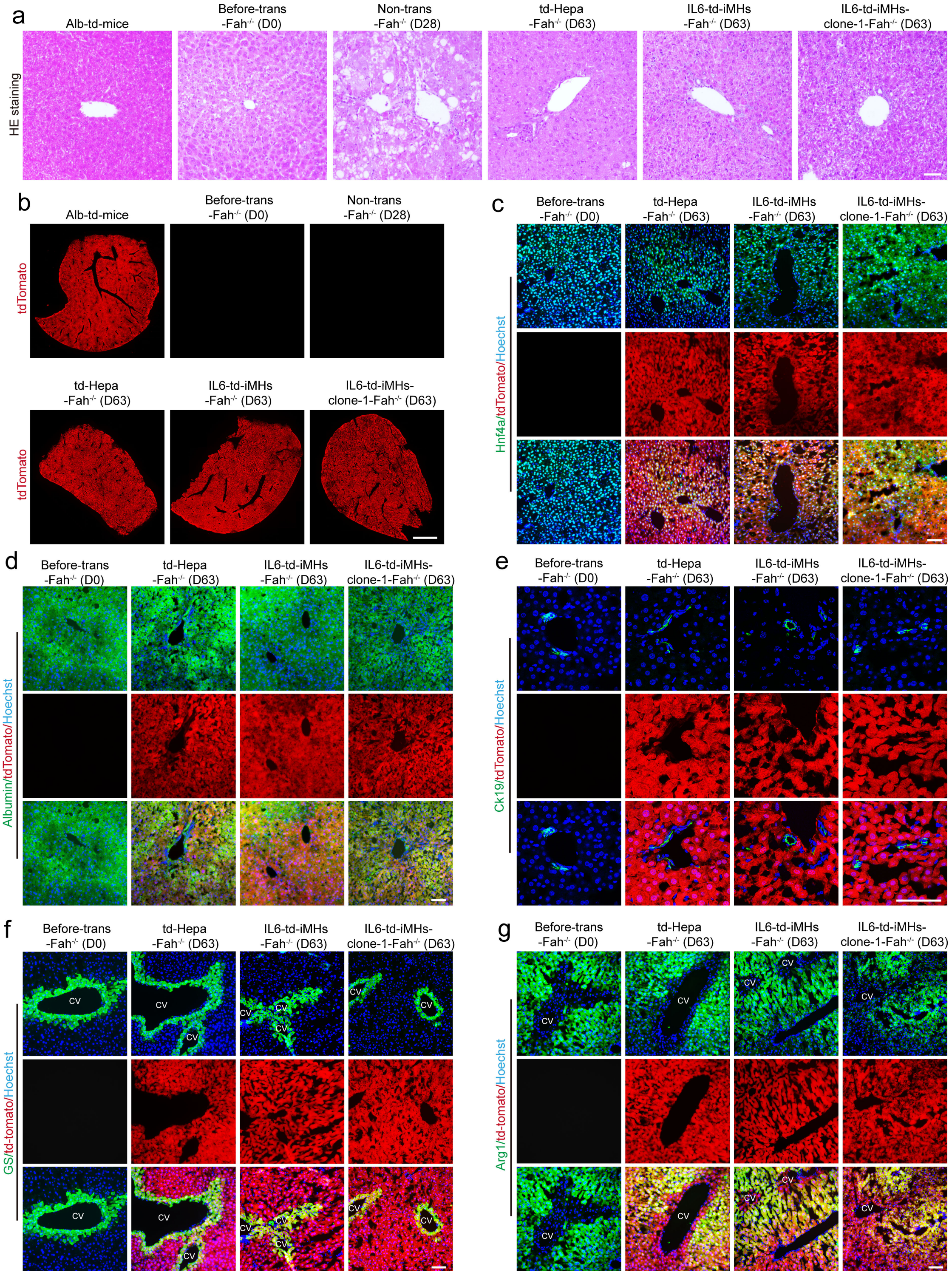

(**a**) Representative images of H&E staining in liver sections from *Fah*$^{-/-}$ mice before NTBC withdrawal (D0, n=2 mice), 28 days after NTBC withdrawal without transplantation (n=3 mice), or 63 days after NTBC withdrawal but receiving td-Hepa (n=3 mice), IL6-td-iMHs (n=6 mice), or IL6-td-iMHs-clone-1 (n=7 mice). (**b**) Frozen section of the left lateral lobe of the liver from *Fah*$^{-/-}$ mice before NTBC withdrawal

(D0), 28 days after NTBC withdrawal without transplantation, or 63 days after NTBC withdrawal but receiving td-Hepa, IL6-td-iMHs, or IL6-td-iMHs-clone-1. The liver of Alb-td-mice was used as control. Scale bars represent 2 mm. (**c-g**) Immunofluorescence staining of hepatic markers Hnf4α (green), Albumin (green), GS (green), Arg1 (green), and cholangiocytes maker Ck19 (green) in frozen liver sections of $Fah^{-/-}$ mice before or 63 days after transplantation. Nuclei were stained with Hoechst 33342 (blue). Scale bars represent 100 μm.

Supplementary Fig. 9 Characterization of hepatocytes isolated from the *Fah*$^{-/-}$ mice transplanted with IL6-td-iMHs for 2 months

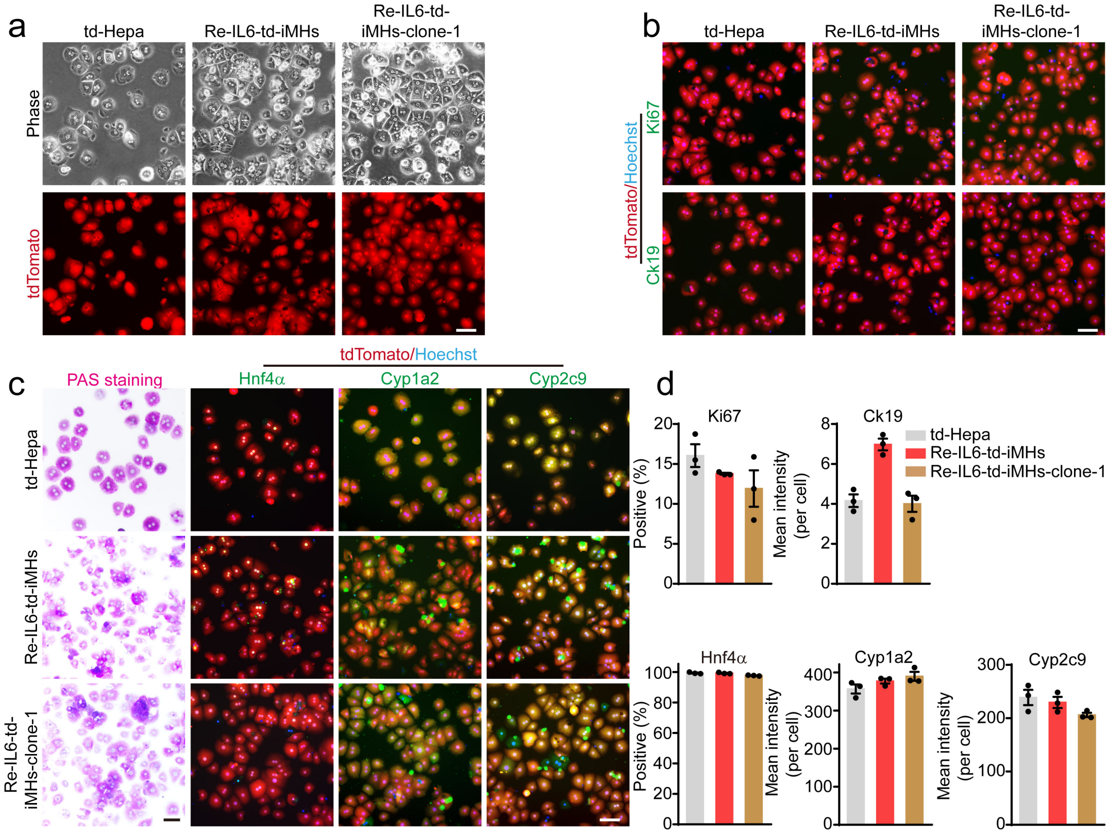

(**a**) Representative morphology and fluorescence images of hepatocytes isolated from *Fah*$^{-/-}$ mice receiving IL6-td-iMHs and IL6-td-iMHs-clone-1 for 63 days after NTBC withdrawal (n=3 mice in each group). Td-Hepa isolated from Alb-td mice were used as control. (**b**) Immunofluorescence staining of cell cycle marker Ki67 (green) and hepatic progenitor cell marker Ck19 (green) in (**a**). (**c**) Representative images of PAS staining and immunofluorescence staining of hepatic markers Hnf4α (green), Cyp1a2 (green) and Cyp2c9 (green) in cells in (**a**). Nuclei were stained with Hoechst 33342 (blue). (**d**) Statistical data of the intensity of Ck19, Cyp1a2 and Cyp2c9, and percent of Ki67 and Hnf4α staining in (**b** and **c**) (n=3 independent experiments). All data are shown as Means ± SEM. Source data are provided in a Source data file. Scale bars represent 100 μm.

Supplementary Fig. 10 Repopulation of Fah$^{-/-}$ liver by long-term cultured IL6-iMHs

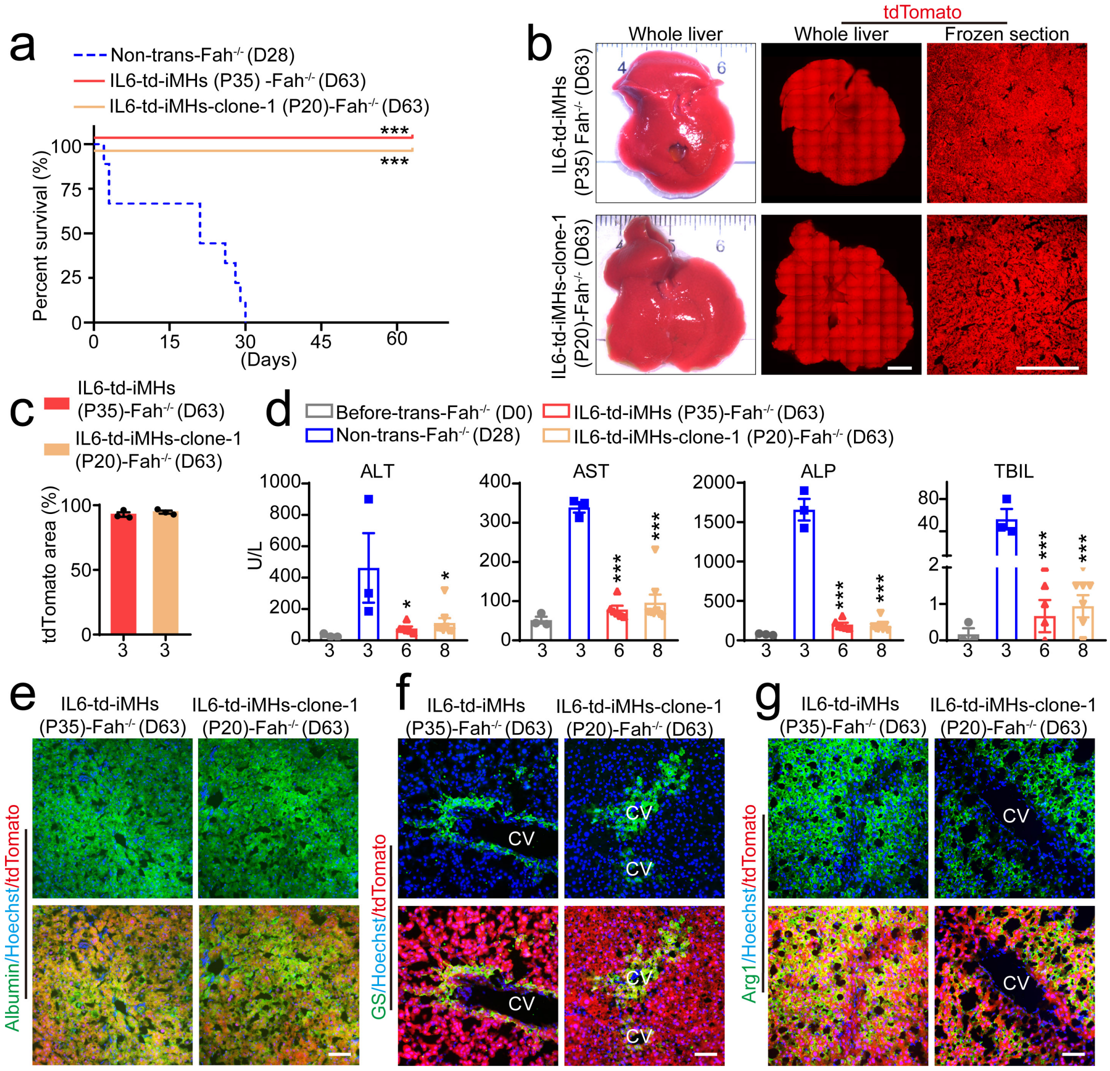

(**a**) Survival curves of *Fah*$^{-/-}$ mice (no NTBC) receiving IL6-td-iMHs (P35, n=6) and IL6-td-iMHs-clone-1 (P20, n=8) or vehicle (Non-trans, n=9, cited from Fig. 4c), ***P< 0.001 (Log-rank test). (**b**) Images of the whole livers (left), fluorescent images of the whole livers (middle, scale bars represent 5 mm) and frozen sections of liver (right, scale bars represent 1 mm) 63 days after IL6-td-iMHs (P35, n=2 mice), or IL6-td-iMHs-clone-1 (P20, n=2 mice) transplantation (no NTBC). (**c**) Quantitative analysis of tdTomato positive areas in liver (n=3 mice per group). (**d**) Serum levels of ALT, AST, ALP and TBIL in the *Fah*$^{-/-}$ mice after transplantation (n=3~8 mice per group, the numbers of the animals were listed under the bars). Data are shown as Means ± SEM. *P < 0.05, ***P< 0.001, vs non-trans (two-tailed unpaired Student's t test). Exact P

values are provided in a Source data file. The data of *Fah*$^{-/-}$ mice before NTBC withdrawal (D0), 28 days after vehicle transplantation (no NTBC) were cited from Fig. 4f. (**e-g**) Immunofluorescence staining of Albumin (green), GS (green) and Arg1 (green) in frozen liver sections of *Fah*$^{-/-}$ mice 63 days after transplantation (n=3 mice). Nuclei were stained with Hoechst 33342 (blue). Source data and exact P values are provided in a Source data file. Scale bars represent 100 μm.

Supplementary Fig. 11 Sorting of hepatocytes with different ploidy

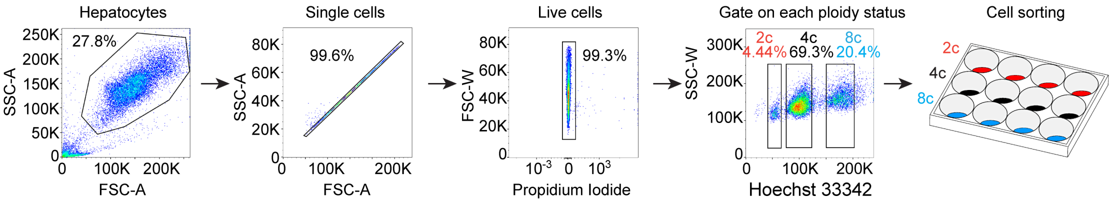

Schematic of the experimental strategy for the FACS sorting of diploid (2c), tetraploid (4c) and octaploid (8c) hepatocytes.

Supplementary Fig. 12 Karyotypes analysis of IL6-iHPCs

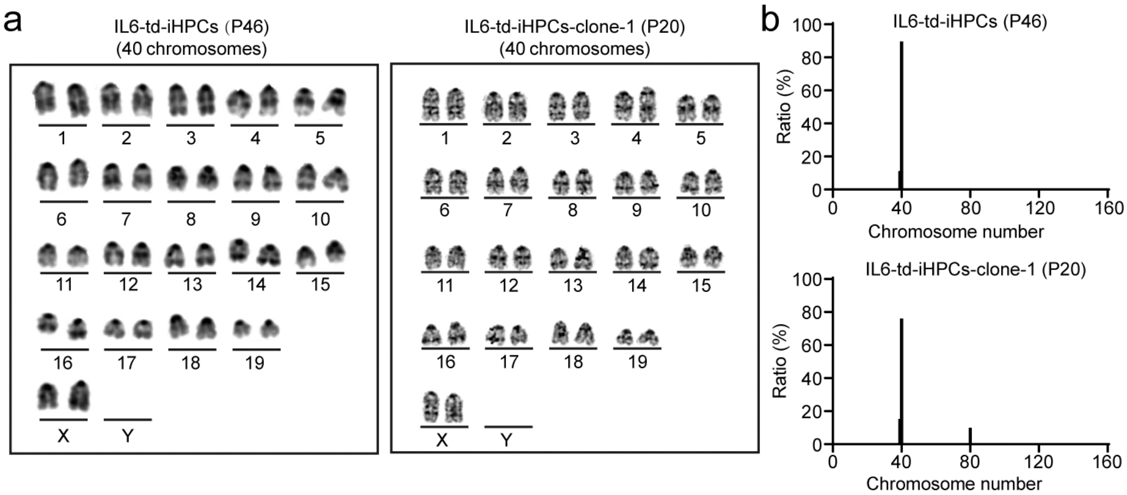

(**a**, **b**) Representative karyotype images (**a**) and statistical analysis of chromosome numbers (**b**) of IL6-td-iHPCs (P46) (n=120 cells) and IL6-td-iHPCs-clone-1 (P20) (n=115 cells).

Supplementary Fig. 13 Involvement of Stat3 signaling in IL6 mediated hepatocyte growth

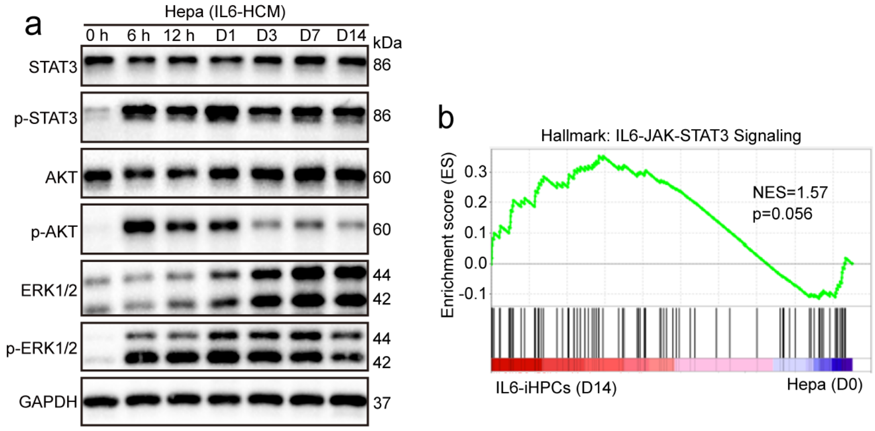

(**a**) Western blotting analysis of STAT3, p-STAT3, AKT, p-AKT, ERK1/2 and p-ERK1/2 in hepatocytes cultured in IL6-HCM for 6 hours to 14 days. GAPDH was used as loading control. Representative blot from 3 independent experiments is shown. (**b**) Gene Set Enrichment Analysis (GSEA) was performed to assess the difference of IL6-iHPCs (D14) and primary hepatocytes using IL6-JAK-STAT3 signature. Normalized enrichment score (NES) reflects the degree of over-representation for each group at the peak of the entire set. P value is calculated using GSEA empirical phenotype-based permutation test, two-sided, and no adjustments were made for multiple comparisons.

Supplementary Fig. 14 Screening of TFs involved in IL6-mediated hepatocyte to iHPC reprogramming

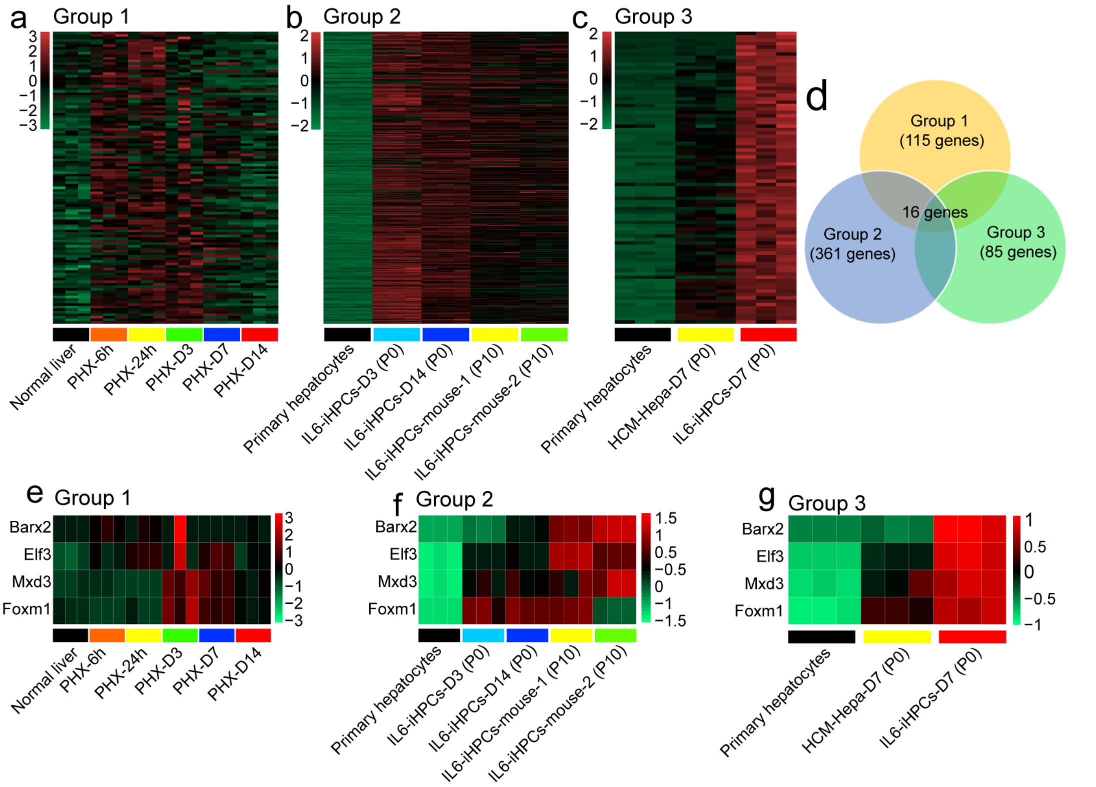

(**a**) Transcriptomic analysis of normal liver and livers post 2/3 partial hepatectomy (PHX) at different time points. Gene expression was compared between PHX-6 h and normal, PHX-24 h and normal, or PHX-D3 and normal. Genes with ≥2-fold changes ($p < 0.05$) from any of the above comparisons were selected (Group 1, 115 genes). (**b**) Transcriptomic analysis of primary hepatocytes, IL6-iHPCs-D3 (P0), IL6-iHPCs-D14 (P0), IL6-iHPCs-mouse-1 (P10) and IL6-iHPCs-mouse-2 (P10). Gene expression was compared between IL6-iHPCs-D14 (P0) and primary hepatocytes, IL6-iHPCs-mouse-1 (P10) and primary hepatocytes, or IL6-iHPCs-mouse-2 (P10) and primary hepatocytes. Genes with ≥2-fold changes ($p < 0.05$) in all of the above comparisons were selected (Group 2, 361 genes). (**c**) Gene expression was compared between IL6-iHPCs-D7 (P0) and HCM-Hepa-D7 (P0), IL6-iHPCs-D7 (P0) and primary hepatocytes. Genes with ≥1.5-fold changes ($p < 0.05$) in all of the above comparisons were selected (Group 3, 85 genes). (**d**) Venn diagram for the selection of TFs. 16 TFs were identified. (**e-g**) Representative genes (*Barx2, Elf3, Mxd3 and FoxM1*) are indicated in group 1, 2, 3. Each element represents the log 2 (normalized expression), as scaled by the corresponding color legends. Three technical replicates for all groups.

Supplementary Fig. 15 Uncropped scans of the immunoblots in Fig. 6a and Supplementary Fig. 11a

Fig. 6a

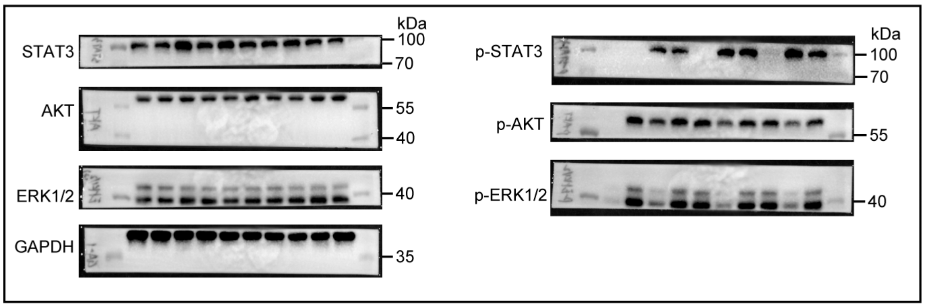

Supplementary. Fig. 11a

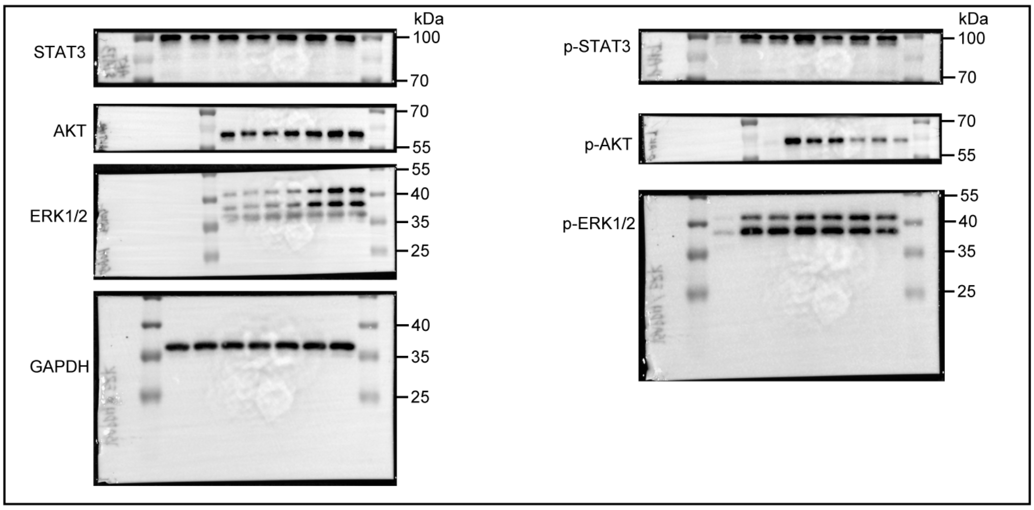

Supplementary Table 1：Reported methods of primary hepatocytes expansion

Cell type	Culture format	Expansion medium/Time	Expansion chemicals/cytokines	Maturation medium/Time	Passages in vitro	Latest passage used to access differentiation and repopulation capacity	Support single cell-derived clones	Ref
Primary hepatocytes (Mice; Rat)	2D	DMEM/F12, 2.4 g/L NaHCO3, L-glutamine, 5 mM HEPES, 30 mg/L L-proline, 0.05% BSA, 10 ng/mL EGF, ITS-X, 10^{-7} M Dex, 10 mM nicotinamide, 1 mM ascorbic acid-2, antibiotic/antimycotic solution;14-16 days	10 μM Y27632, 0.5 μM A-83-01, 3 μM CHIR99021;	DMEM/F12, 2.4 g/L NaHCO3, L-glutamine, 5 mM HEPES, 30 mg/L L-proline, 0.05% BSA, 10 ng/mL EGF, ITS, 10^{-6} M Dex, 10 mM nicotinamide, 1 mM ascorbic acid-2, 10 μM Y27631, 0.5 μM A-83-01, 3 μM CHIR99021, 20 ng/mL OSM; 8 days	Rat: At least 26 passages, doubling time =14.7 ± 1.1 hr; Mice: At least 20 passages	Rat: differentiation capacity was measured at P ≥15. In vivo repopulation capacity was measured for clones with passages not specified. Mice: differentiation capacity was measured at P11-12.	Rat (Yes); Mice (Not described)	1
Primary hepatocytes (Mice)	2D	DMEM/F12, N2 or ITS, 20 ng/mL EGF, 20 ng/mL HGF; 7 days	10 μM Y27632, 3 μM CHIR99021, 1 μM S1P, 5 μM LPA, 1 μM A83-01;	DMEM/F12, N2 or ITS, 10 μM DAPT, 20 ng/mL OSM, 10 μM Dex, 10 μM SB431542; 12-21 days	At least 30 passages, doubling time=15-20 hr	Differentiation and in vivo repopulation capacity were measured at passages not specified.	Not described	2
Primary hepatocytes (Mice)	2D	DMEM/F12, 1% FBS, ITS, 10^{-7} M Dex, 10 mM nicotinamide, 50 μM β-mercaptoethanol,	4 μM A-83-01, 3 μM CHIR99021	DMEM/F12, 1%FBS, ITS, 10^{-5} M Dex, 10 mM nicotinamide, 50 μM β-mercaptoethanol, 20 ng/mL EGF, 20 ng/mL HGF, 4 μM A-83-01, 3	At least 23 passages, doubling time=35.42±	Differentiation was measured at P23. In vivo repopulation capacity was measured with single cell-	Yes	3

		20 ng/mL EGF, 20 ng/mL HGF, 1% penicillin/streptomycin; 8 days		μM CHIR99021, 20 ng/mL OSM, 1% penicillin/streptomycin; 8 days	0.8 hr	derived clone with passage not specified.		
Primary hepatocytes (Mice)	3D	William's E medium, 1% Glutamax, 1% Non-Essential Amino Acids, 0. 2% normocin, 2% B27, 1% N2, 10 mM nicotinamide, 1.25 mM N-acetylcysteine, 25 ng/mL EGF, 50 ng/mL HGF, 1% penicillin/streptomycin; 14 days;	10 μM Y27632, 1 μM A-83-01, 3 μM CHIR99021, 100 ng/mL TNFa, 50 ng/mL noggin	William's E medium, 1% Glutamax, 1% Non-Essential Amino Acids, 0. 2% normocin, 2% B27, 1% N2, 10 mM nicotinamide, 1.25 mM N-acetylcysteine, 25 ng/mL EGF, 50 ng/mL HGF, 1% penicillin/streptomycin, (condition 1: 10 μM Y27632, 1 μM A-83-01, 3 μM Dex; condition 2: 3 μM CHIR99021, 3 μM Dex); 3-5 days	About 14 passages, 7 months	Functional properties are retained in 7 months cultures (P14). In vivo repopulation capacity was measured with the cells cultured for 105 days.	Not described	4
Primary hepatocytes (Human)	2D	DMEM/F12, 1% FBS, ITS, 10^{-7} M Dex, 10 mM nicotinamide, 20 ng/mL EGF, 20 ng/mL HGF; 15 days	4 μM A-83-01, 3 μM CHIR99021	DMEM/F12, 1%FBS, ITS, 10^{-7} M Dex, 10 mM nicotinamide, 20 ng/mL EGF, 20 ng/mL HGF, 20 ng/mL OSM; 8 days	10 passages, doubling time=37-47 hr	Differentiation was measured at P10. In vivo repopulation was measured at P6	Not described	5
Primary hepatocytes (Human)	2D	Advanced DMEM/F12, N2, B27, 1 mM sodium pyruvate, 10 μg/mL ascorbic acid, 20 ng/mL EGF, 20 ng/mL HGF; 6-12 days	10 μM Y27632, 3 μM CHIR99021, 1 μM A-83-01, 1 μM S1P, 5 μM LPA	Advanced DMEM/F12, N2, B27, 10 μM DAPT, 20 ng/mL OSM, 10 μM Dex 10 μM SB431542; 6 days	10 passages, doubling time=24.7± 1.4 hr	Differentiation was measured at P10. In vivo repopulation was measured at P5.	Not described	6

Primary hepatocytes (Human)	2D	Advanced DMEM/F12, N2, B27 (minus vitamin A), 1mM N-acetyl-cysteine, 10 mM nicotinamide, 50 ng/mL EGF, 25 ng/mL HGF, 1% FBS, 1% penicillin/streptomycin; 5 days	10 nM Human [Leu15]-gastrin I, 2 ng/mL FGF10, 5 μM A83-01, 10 μM Y27632, 50 ng/mL Wnt3a protein	Advanced DMEM/F12, N2, B27 (minus vitamin A), 1mM N-acetyl-cysteine, 10 mM nicotinamide, 2 ng/mL FGF10, 50 ng/mL EGF, 25 ng/l HGF, 10 nM Human [Leu15]-gastrin I, 1% FBS, 5 μM A83-01, 10 μM Y27632, 50 ng/mL Wnt3a protein, 5 μM Forskolin, 1 μM Dex, 20 ng/mL OSM, 1% FBS, 1% penicillin/streptomycin; 10 days	(Hypoxia) 8 passages	Differentiation was measured at P8. Repopulation was measured at P6.	Not described	7
Primary hepatocytes (mice)	3D	AdDMEM/F12, 15% RSPO1 conditioned medium, B27 (minus vitamin A), 50 ng/mL EGF, 1.25 mM N-acetylcysteine, 10 mM Nicotinamide, 25 ng/mL HGF; 14 days	10 nM gastrin, 100 ng/mL FGF7, 100 ng/mL FGF10, 3 μM CHIR99021, 2 μM A83-01, 10 μM Y27632	None	7 passages estimated from Figure 3B	Not described.	Not described	8
Primary hepatocytes (Human fetal, adult)	3D	AdDMEM/F12, 15% RSPO1 conditioned medium, B27 (minus vitamin A), 50 ng/mL EGF, 1.25 mM N-acetylcysteine, 10 mM Nicotinamide, 50 ng/mL HGF; 9-10	10 nM gastrin, 100 ng/mL FGF7, 100 ng/mL FGF10, 2 μM A83-01, 10 μM Y27632, 20 ng/mL TGFa	For fetal organoids: AdDMEM/F12, 15% RSPO1 conditioned medium,B27 (minus vitamin A), 50 ng/mL EGF, 1.25 mM N-acetylcysteine, 10 mM Nicotinamide, 50 ng/mL HGF, 10 nM gastrin, 100 ng/mL FGF7, 100 ng/mL FGF10, 2 μM A83-01, 10	Fetal: 28 passages; adult: 2–2.5 months	Human fetal hepatocyte organoids：Differentiation was measured at P22. Repopulation was measured at P16. Human adult hepatocyte organoids：In vivo	Not described	8

		days		µM Y27632, 20 ng/mL TGFa, 1 µM Dex, 10 ng/mL OSM;		repopulation capacity was measured (passage was not specified)		
Primary hepatocytes (mice)	2D	DMEM/F12, ITS, 10^{-7} M Dex, 20 ng/mL EGF, 20 ng/mL HGF; 14 days	30 ng/mL IL6	DMEM/F12, ITS, 10^{-6} M Dex, 20 ng/mL OSM; 7days	At least 46 passages, doubling time=13-20 hr	Differentiation was measured at P5, P10, P20, P30 and P35. Repopulation was measured at P10, P20 and P35.	Yes	here

Supplementary Table 2：Primer sequences for quantitative RT-PCR Analysis

Gene	Forward	Reverse
Afp	TGCAGAAACACATCGAGGAGAG	GCTTCACCAGGTTAATGAGAAGCT
Cxcr4	TTGTCCACGCCACCAACAGTCA	TGAAACACCACCATCCACAGGC
Cd44	CACCATTGCCTCAACTGTGC	TTGTGGGCTCCTGAGTCTGA
Cd133	CTCCCATCAGTGGATAGAGAACT	ATACCCCCTTTTGACGAGGCT
Cldn3	TCACGGCGCAGATCACCT	ACCAACGGGTTATAGAAATCCCT
Ck8	AGATGAACCGCAACATCAACC	TCAATCTTCTTCACAACCACAGC
Ck19	GTCCTACAGATTGACAATGC	CACGCTCTGGATCTGTGACAG
Fn14	GTGTTGGGATTCGGCTTGGT	GTCCATGCACTTGTCGAGGTC
Sox9	CACCCCGATTACAAGTACCAG	TGCTCAGTTCACCGATGTCCA
Epcam	CTGGGAGGAGGATAAAGC	AGAAGAATGGAACAGGGAC
Bcl2	GTCGCTACCGTCGTGACTTC	CAGACATGCACCTACCCAGC
Cdk1	AGAAGGTACTTACGGTGTGGT	GAGAGATTTCCCGAATTGCAGT
CyclinB1	AAGGTGCCTGTGTGTGAACC	GTCAGCCCCATCATCTGCG
Cdc20	TTCGTGTTCGAGAGCGATTTG	CAGACATGCACCTACCCAGC
Albumin	CCACTGTTGAAGAAAGCCCA	CAGATAGTCTTCCACACAAGGCA
G6p	GCGCAGCAGGTGTATACTATGT	ATCAACTCAACCTGGGATGG
Hnf4a	CAGGGGCTTGGGTGGCATCCT	CTGCAGGAGCGCGTTGATGGA
Fah	CACGAGACATCCAGCAAT	GGTTCCAGAAGCCAAGAG
Aat	AATGGAAGAAGCCATTCGAT	AAGACTGTAGCTGCTGCAGC
Trf	CCCACTCAAATGTGCTCCGAAC	ACCGACAGATTGCATGTACTCC
Ttr	CTCACCACAGATGAGAAG	GGCTGAGTCTCTCAATTC
Foxa1	CACAGGGTTGGATGGTTGTGT	GTACGCCATGGGACTCATGCA
Foxa2	GGAGCAGCGGCCAGCGAGTTA	TCTGCTGGATGGCCATGGTGA
Foxa3	TGTAGAGAGACCGAAGCAC	AGGTCCATGATCCATTGGTA
Cyp1a1	CAATGAGTTTGGGGAGGTTACTG	CCCTTCTCAAATGTCCTGTAGTG
Cyp1a2	AGTACATCTCCTTAGCCCCAG	GGTCCGGGTGGATTCTTCAG
Cyp3a11	TGAGGCAGAAGGCAAAGAAA	GGTATTCCATCTCCATCACA
Cyp3a13	GACGATTCTTGCTTACCAGAAGG	CCGGTTTGTGAAGGTAGAGTAAC
Cyp3a16	TGTCCTTGTCAGTAGCACTCT	TGTGATCTCGATTTCAGAAAGGG
Cyp3a41	AAAGCCGCCTCGATTCTAAGC	ACTACATCCCGTGGTACAACC
Cyp2b10	AAAGTCCCGTGGCAACTTCC	TTGGCTCAACGACAGCAACT
Cyp2c38	CACGGCCCATTGTTGTATTGC	TGAGTGTGAAACGTCTTGTCTCT
Cyp2d22	CAGTGGTTGTACTAAATGGGCT	GCTAGGACTATACCTTGAGAGCG
Barx2	GATGGTCCTTAAAGGTGGACAG	TGGGCTCCTGGGTATCACAG
FoxM1	CTGATTCTCAAAAGACGGAGGC	TTGATAATCTTGATTCCGGCTGG
Mxd3	GAGGCAGAGCACGGTTATG	TGTAGTGTATCGGGTACAGTCAA
Elf3	GCTGCCACCTGTGAGATCAG	GTGCCAAAGGTAGTCGGAGG
GAPDH	AGGTCGGTGTGAACGGATTTG	TGTAGACCATGTAGTTGAGGTCA

Supplementary Table 3：Short hairpin RNAs oligonucleotide used

Name	Forward	Reverse
Scramble	CCGGCCTAAGGTTAAGTCGCCCTC<br>GCTCGAGCGAGGGCGACTTAACCT<br>TAGGTTTTTG	GGCCGGATTCCAATTCAGCGGGAGC<br>GAGCTCGCTCCCGCTGAATTGGAAT<br>CCAAAAAC
Barx2 shRNA	CCGGGCTGCAAGTGAAGACTTGGT<br>ACTCGAGTACCAAGTCTTCACTTG<br>CAGCTTTTTG	AATTCAAAAAGCTGCAAGTGAAGA<br>CTTGGTACTCGAGTACCAAGTCTTC<br>ACTTGCAGC
FoxM1 shRNA	CCGGGCTCCATAGAAATGTGACCA<br>TCTCGAGATGGTCACATTTCTATGG<br>AGCTTTTTG	AATTCAAAAAGCTCCATAGAAATGT<br>GACCATCTCGAGATGGTCACATTTCT<br>ATGGAGC
Elf3 shRNA	CCGGCTTGGTGTTGACCCTGAACA<br>ACTCGAGTTGTTCAGGGTCAACAC<br>CAAGTTTTTG	AATTCAAAAACTTGGTGTTGACCCT<br>GAACAACTCGAGTTGTTCAGGGTCA<br>ACACCAAG
Mxd3 shRNA	CCGGCCACATGTTGAAGAGACTAA<br>ACTCGAGTTTAGTCTCTTCAACATG<br>TGGTTTTTG	AATTCAAAAACCACATGTTGAAGAG<br>ACTAAACTCGAGTTTAGTCTCTTCA<br>ACATGTGG

REFERENCES

1 Katsuda, T. *et al.* Conversion of Terminally Committed Hepatocytes to Culturable Bipotent Progenitor Cells with Regenerative Capacity. *Cell Stem Cell* **20**, 41-55, doi:10.1016/j.stem.2016.10.007 (2017).

2 Wu, H. *et al.* Reversible transition between hepatocytes and liver progenitors for in vitro hepatocyte expansion. *Cell Res* **27**, 709-712, doi:10.1038/cr.2017.47 (2017).

3 Kim, Y. *et al.* Adenine base editing and prime editing of chemically derived hepatic progenitors rescue genetic liver disease. *Cell Stem Cell* **28**, 1614-+, doi:10.1016/j.stem.2021.04.010 (2021).

4 Peng, W. C. *et al.* Inflammatory Cytokine TNFalpha Promotes the Long-Term Expansion of Primary Hepatocytes in 3D Culture. *Cell* **175**, 1607-1619 e1615, doi:10.1016/j.cell.2018.11.012 (2018).

5 Kim, Y. *et al.* Small molecule-mediated reprogramming of human hepatocytes into bipotent progenitor cells. *J Hepatol* **70**, 97-107, doi:10.1016/j.jhep.2018.09.007 (2019).

6 Fu, G. B. *et al.* Expansion and differentiation of human hepatocyte-derived liver progenitor-like cells and their use for the study of hepatotropic pathogens. *Cell Res* **29**, 8-22, doi:10.1038/s41422-018-0103-x (2019).

7 Zhang, K. *et al.* In Vitro Expansion of Primary Human Hepatocytes with Efficient Liver Repopulation Capacity. *Cell Stem Cell* **23**, 806-819 e804, doi:10.1016/j.stem.2018.10.018 (2018).

8 Hu, H. L. *et al.* Long-Term Expansion of Functional Mouse and Human Hepatocytes as 3D Organoids. *Cell* **175**, 1591-+, doi:10.1016/j.cell.2018.11.013 (2018).
