# Supplementary material for: IL6 supports long-term expansion of hepatocytes in vitro
Source: Nat Commun. 2022 Nov 29;13:7345. doi: 10.1038/s41467-022-35167-8 (PMC9708838; doi:10.1038/s41467-022-35167-8)
Supplement: Supplementary file 2 — Reporting Summary [file 41467_2022_35167_MOESM2_ESM.pdf]

nature portfolio

Corresponding author(s): Xin Xie, Ph. D.

Last updated by author(s): Nov 12, 2022

Reporting Summary

Nature Portfolio wishes to improve the reproducibility of the work that we publish. This form provides structure for consistency and transparency in reporting. For further information on Nature Portfolio policies, see our Editorial Policies and the Editorial Policy Checklist.

Statistics

For all statistical analyses, confirm that the following items are present in the figure legend, table legend, main text, or Methods section.

n/a	Confirmed	
☐	☒	The exact sample size (*n*) for each experimental group/condition, given as a discrete number and unit of measurement
☒	☐	A statement on whether measurements were taken from distinct samples or whether the same sample was measured repeatedly
☐	☒	The statistical test(s) used AND whether they are one- or two-sided<br>*Only common tests should be described solely by name; describe more complex techniques in the Methods section.*
☒	☐	A description of all covariates tested
☒	☐	A description of any assumptions or corrections, such as tests of normality and adjustment for multiple comparisons
☐	☒	A full description of the statistical parameters including central tendency (e.g. means) or other basic estimates (e.g. regression coefficient) AND variation (e.g. standard deviation) or associated estimates of uncertainty (e.g. confidence intervals)
☐	☒	For null hypothesis testing, the test statistic (e.g. *F*, *t*, *r*) with confidence intervals, effect sizes, degrees of freedom and *P* value noted<br>*Give P values as exact values whenever suitable.*
☒	☐	For Bayesian analysis, information on the choice of priors and Markov chain Monte Carlo settings
☒	☐	For hierarchical and complex designs, identification of the appropriate level for tests and full reporting of outcomes
☒	☐	Estimates of effect sizes (e.g. Cohen's *d*, Pearson's *r*), indicating how they were calculated

Our web collection on statistics for biologists contains articles on many of the points above.

Software and code

Policy information about availability of computer code

Data collection	Applied Agilent Mx3000p Real-Time PCR system; Olympus 1X2-ILL100 Microscopy; Bio Rad CHEMI DOC M Chemiluminescent Image Systme; BD FACS Aria Ⅲ system.
Data analysis	Graphs plotted and statistical calculations in Prism 8 version 8.0.1. The Immunofluorescence staining and positive cell percentage were analyzed using ImageJ 1.46 r. Heatmap and correlation map were drawn by R software (v4.2.1, https://www.r-project.org)

For manuscripts utilizing custom algorithms or software that are central to the research but not yet described in published literature, software must be made available to editors and reviewers. We strongly encourage code deposition in a community repository (e.g. GitHub). See the Nature Portfolio guidelines for submitting code & software for further information.

Data

Policy information about availability of data

All manuscripts must include a data availability statement. This statement should provide the following information, where applicable:

- Accession codes, unique identifiers, or web links for publicly available datasets
- A description of any restrictions on data availability
- For clinical datasets or third party data, please ensure that the statement adheres to our policy

Source data are provided with this paper. The microarray data are available at the NCBI Gene Expression Omnibus with accession number GSE215423 (https://www.ncbi.nlm.nih.gov/geo/query/acc.cgi?acc=GSE215423).

Human research participants

Policy information about studies involving human research participants and Sex and Gender in Research.

Reporting on sex and gender	Do not have any sex and gender research in this literature
Population characteristics	Do not have any human population characteristics in this literature
Recruitment	Do not have any participants recruiting.
Ethics oversight	Do not have any human research in this literature.

Note that full information on the approval of the study protocol must also be provided in the manuscript.

Field-specific reporting

Please select the one below that is the best fit for your research. If you are not sure, read the appropriate sections before making your selection.

☒ Life sciences ☐ Behavioural & social sciences ☐ Ecological, evolutionary & environmental sciences

For a reference copy of the document with all sections, see nature.com/documents/nr-reporting-summary-flat.pdf

Life sciences study design

All studies must disclose on these points even when the disclosure is negative.

Sample size	Sample size was choosen based on previous experiments (DOI: 10.1016/j.stem.2016.10.007; DOI: 10.1016/j.stem.2021.04.010; DOI:10.1038/cr.2017.47) or based on exploratory experiments to ensure the possibility of statistical analysis and to minimize the use of experimental animals based on the 3R principles.
Data exclusions	No data were excluded from the analyses.
Replication	All the experiments and data shown in this manuscript have been repeated independently at least three times with similar results as indicated in the legends.
Randomization	Allocation was random.
Blinding	Blinding was not done as most of the experiments were carried out by a single person and the data were collected and analyzed by the same person. It was not feasible during the course of the study to have at least two individuals for each experiment. No behavioral experiments were included in this study, no data was excluded in this study and all analyses were performed in a quantitative and objective way.

Reporting for specific materials, systems and methods

We require information from authors about some types of materials, experimental systems and methods used in many studies. Here, indicate whether each material, system or method listed is relevant to your study. If you are not sure if a list item applies to your research, read the appropriate section before selecting a response.

Materials & experimental systems

n/a	Involved in the study	
☐	☒	Antibodies
☐	☒	Eukaryotic cell lines
☒	☐	Palaeontology and archaeology
☐	☒	Animals and other organisms
☒	☐	Clinical data
☒	☐	Dual use research of concern

Methods

n/a	Involved in the study	
☒	☐	ChIP-seq
☐	☒	Flow cytometry
☒	☐	MRI-based neuroimaging

Antibodies

Antibodies used	Ki67 (#9129S), STAT3 (#9139S), p-STAT3 (#9145S), Akt (#9272S) , p-Akt (#4060S), ERK1/2 (#4695S), p-ERK1/2 (#4370S), GAPDH (#2118S), Anti-rabbit IgG, HRP-linked (#7074S), Anti-mouse IgG, HRP-linked (#7076S), antibodies were purchased from Cell Signaling Technologies (Danvers, MA, USA). CyclinD1 (#ab134175), Cytokeratin 19 (#ab52625), Hnf4a(#ab181604), Cyp1a2 (#ab22717), Cyp2c9 (#ab4236), GS (#ab49873), Arg1 (#ab96183) antibodies were purchased from Abcam (Cambridge, MA, USA). Sox9

(#Millipore-ab5535) antibody was purchased from Merck Millipore (Billerica, MA, USA). Albumin (#A90-460 234A) antibody was purchased from Bethyl Laboratories (Montgomery, TX, USA). Fah (#GTX114400) antibody was purchased from GeneTex (San Antonio, TX, USA). Alexa Fluor 555 Goat Anti-mouse IgG (#A32727), Alexa Fluor 555 Goat Anti-rabbit IgG (#A32732), Alexa Fluor 488 Rabbit Anti-goat IgG (#A27012), Alexa Fluor 488 Goat Anti-mouse IgG (#A32723) and Alexa Fluor 488 Goat Anti-rabbit IgG (#A11034) antibodies were purchased from Invitrogen (Carlsbad, CA, USA). IL6 neutralizing antibody (MAB406-SP), IgG (6-001-A) were purchased from R&D Systems (Minneapolis, MN, USA).

Validation

Rabbit anti-Ki67 Cell Signaling Technologies #9129S 1:200
https://www.cellsignal.cn/products/primary-antibodies/ki-67-d3b5-rabbit-mab/9129
Mouse anti-Stat3 Cell Signaling Technologies #9139S 1:1000
https://www.cellsignal.cn/products/primary-antibodies/stat3-124h6-mouse-mab/9139
Rabbit p-STAT3 Cell Signaling Technologies #9145S 1:1000
https://www.cellsignal.cn/products/primary-antibodies/phospho-stat3-tyr705-d3a7-xp-rabbit-mab/9145
Rabbit anti-Akt Cell Signaling Technologies #9272S 1:1000
https://www.cellsignal.cn/products/primary-antibodies/akt-antibody/9272
Rabbit p-Akt Cell Signaling Technologies #4060S 1:1000
https://www.cellsignal.cn/products/primary-antibodies/phospho-akt-ser473-d9e-xp-rabbit-mab/4060
Rabbit anti-Erk1/2 Cell Signaling Technologies #4695S 1:1000
https://www.cellsignal.cn/products/primary-antibodies/p44-42-mapk-erk1-2-137f5-rabbit-mab/4695
Rabbit p-Erk1/2 Cell Signaling Technologies #4370S 1:1000
https://www.cellsignal.cn/products/primary-antibodies/phospho-p44-42-mapk-erk1-2-thr202-tyr204-d13-14-4e-xp-rabbit-mab/4370
Rabbit anti-GAPDH Cell Signaling Technologies #2118S 1:8000
https://www.cellsignal.cn/products/primary-antibodies/gapdh-14c10-rabbit-mab/2118
Goat anti-rabbit IgG, HRP-linked Antibody Cell Signaling Technologies #7074S 1:10000
https://www.cellsignal.cn/products/secondary-antibodies/anti-rabbit-igg-hrp-linked-antibody/7074
Horse anti-mouse IgG, HRP-linked Antibody Cell Signaling Technologies #7076S 1:10000
https://www.cellsignal.cn/products/secondary-antibodies/anti-mouse-igg-hrp-linked-antibody/7076
Rabbit anti-CyclinD1 Abcam #ab134175 1:200
https://www.abcam.cn/cyclin-d1-antibody-epr2241-c-terminal-ab134175.html
Rabbit anti- Cytokeratin 19 Abcam #ab52625 1:200
https://www.abcam.cn/cytokeratin-19-antibody-ep1580y-cytoskeleton-marker-ab52625.html
Rabbit anti-Hnf4a Abcam #ab181604 1:200
https://www.abcam.cn/hnf-4-alpha-antibody-epr16885-chip-grade-ab181604.html
Mouse anti-Cyp1a2 Abcam #ab22717 1:200
https://www.abcam.cn/cytochrome-p450-1a2-antibody-d15-16vii-f10f12-ab22717.html
Rabbit anti- Cyp2c9 Abcam #ab4236 1:200
https://www.abcam.cn/products/primary-antibodies/cyp2c9-antibody-ab4236.html
Rabbit anti-GS Abcam #ab49873 1:500
https://www.abcam.cn/glutamine-synthetase-antibody-ab49873.html
Rabbit anti-Arg1 Abcam #ab96183 1:200
https://www.abcam.cn/liver-arginase-antibody-ab96183.html
Rabbit anti-Sox9 Merck Millipore # ab5535 1:200
https://www.sigmaaldrich.cn/CN/en/sds/mm/ab5535
Goat anti-Albumin Bethyl Laboratories #A90-234A 1:200
https://www.biomol.com/products/antibodies/primary-antibodies/general/anti-mouse-albumin-cross-adsorbed-a90-234a
Rabbit anti-Fah GeneTex #GTX114400 1:200
https://www.genetex.cn/PDF/Download?catno=GTX114400
Rabbit anti-IL-6 R&D Systems #MAB406-SP
https://resources.rndsystems.com/pdfs/datasheets/mab406.pdf
Rat anti-IgG R&D Systems #6-001-A
https://resources.rndsystems.com/pdfs/datasheets/6-001-a.pdf
Alexa Fluor 555 Goat Anti-mouse IgG Invitrogen #A32727 1:1000
https://www.thermofisher.cn/order/genome-database/dataSheetPdf
Alexa Fluor 555 Goat Anti-rabbit IgG Invitrogen #A32732 1:1000
https://www.thermofisher.cn/order/genome-database/dataSheetPdf
Alexa Fluor 488 Rabbit Anti-goat IgG Invitrogen #A27012 1:1000
https://www.thermofisher.cn/order/genome-database/dataSheetPdf
Alexa Fluor 488 Goat Anti-mouse IgG Invitrogen #A32723 1:1000
https://www.thermofisher.cn/order/genome-database/dataSheetPdf
Alexa Fluor 488 Goat Anti-rabbit IgG Invitrogen #A11008 1:1000
https://www.thermofisher.cn/order/genome-database/dataSheetPdf

Eukaryotic cell lines

Policy information about cell lines and Sex and Gender in Research

Cell line source(s)

HEK 293T (CRL-3216) was obtained from American Type Culture Collection (ATCC).

Authentication	None of the cell lines have been authenticated.
Mycoplasma contamination	PCR experiment was tested for mycoplasma contamination and resulted negative.
Commonly misidentified lines (See ICLAC register)	No commonly misidentified cell lines were used in the study.

Animals and other research organisms

Policy information about studies involving animals; ARRIVE guidelines recommended for reporting animal research, and Sex and Gender in Research

Laboratory animals	All mice were housed under controlled humidity (humidity (60 ± 10%) and temperature (22 ± 1°C) conditions and under 12-hour light/dark cycles.
Wild animals	The study did not involve any wild animals.
Reporting on sex	The study did not involve any sex restriction.
Field-collected samples	Mice were maintained on a 12 hour light/dark cycle with free access to a normal chow diet (Shanghai Laboratory Animal Co. Ltd, China) and water at an accredited animal facility at Shanghai Institutes of Materia Medica.
Ethics oversight	All experimental procedures and protocols were approved by the Institutional Animal Care and Use Committees at the Shanghai Institute of Materia Medica.

Note that full information on the approval of the study protocol must also be provided in the manuscript.

Flow Cytometry

Plots

Confirm that:

- [x] The axis labels state the marker and fluorochrome used (e.g. CD4-FITC).
- [x] The axis scales are clearly visible. Include numbers along axes only for bottom left plot of group (a 'group' is an analysis of identical markers).
- [x] All plots are contour plots with outliers or pseudocolor plots.
- [x] A numerical value for number of cells or percentage (with statistics) is provided.

Methodology

Sample preparation	For flow cytometry analysis of hepatocyte ploidy populations, primary hepatocytes and IL6-iHPCs were fixed and permeabilized with Fixation/Permeabilization buffer (eBioscience™, 005123-43) for 20 min at 4°C, after thorough wash, cells were incubated with 10 ug/mL propidium iodide (containing 0.2 mg/mL RNAase) for 30 min at room temperature, then cells were transferred into the 96 well plate through a 40 um filter membrane.<br>For flow cytometry sorting of 2c, 4c, 8c-hepatocytes, primary hepatocytes were suspended in DMEM/F12 supplemented with 50 ug/mL Hoechst 33342 and 5 uM reserpine and incubated at 37 °C for 10 minutes. Following the addition of 5 ug/mL propidium iodide (Yeasen, 40711ES10).
Instrument	For flow cytometry analysis of hepatocytes ploidy populations, cells were analyzed with flow cytometer (ACEA Novocyte) .<br>For flow cytometry sorting of 2c, 4c, 8c-hepatocytes, cells were analyzed using a FACS Aria III system (BD Biosciences).
Software	The data were collected with NovoExpress software and the statistics data were analyzed with GraphPad Prism 8.
Cell population abundance	200,000 cells per well were plated into corresponding well-plates. After digest, staining, and cell filtration, about 100,000 cells left at each sample. And 30,000 cells were decided to analysis in each sample, with pre-setting of instrument and software. We used hepatocytes to perform flow cytometry assay, the cell type is simple.
Gating strategy	For flow cytometry analysis of hepatocytes ploidy populations, the gating strategy was simple that chosen the obvious population.<br>For flow cytometry sorting of 2c, 4c, 8c-hepatocytes. We stained cells with PI to exclude dead cells, and stained cells with Hoechst to distiguish 2c, 4c, 8c-hepatocytes. The gating strategy was that chosen the obvious population.

- [x] Tick this box to confirm that a figure exemplifying the gating strategy is provided in the Supplementary Information.
